# Supplementary material for: Global distribution and drivers of relative contributions among soil nitrogen sources to terrestrial plants
Source: Nat Commun. 2024 Jul 30;15:6407. doi: 10.1038/s41467-024-50674-6 (PMC11289379; doi:10.1038/s41467-024-50674-6)
Supplement: Supplementary file 1 — Supplementary Information [file 41467_2024_50674_MOESM1_ESM.pdf]

Supplementary Information for

Global distribution and drivers of relative contributions among soil nitrogen sources to terrestrial plants

Chao-Chen Hu[1], Xue-Yan Liu[1]*, Avery W. Driscoll[2], Yuan-Wen Kuang[3], E. N. Jack Brookshire[4], Xiao-Tao Lü[5], Chong-Juan Chen[1], Wei Song[1], Rong Mao[6], Cong-Qiang Liu[1], Benjamin Z Houlton[7]

[1] School of Earth System Science, Tianjin University, Tianjin, China
[2] Department of Soil and Crop Sciences, Colorado State University, Fort Collins, CO, USA
[3] Guangdong Provincial Key Laboratory of Applied Botany and Key Laboratory of Vegetation Restoration and Management of Degraded Ecosystems, South China Botanical Garden, Chinese Academy of Sciences, Guangzhou, China
[4] Department of Land Resources and Environmental Sciences, Montana State University, Bozeman, MT, USA
[5] Erguna Forest-Steppe Ecotone Research Station, Institute of Applied Ecology, Chinese Academy of Sciences, Shenyang, China
[6] Key Laboratory of National Forestry and Grassland Administration On Forest Ecosystem Protection and Restoration of Poyang Lake Watershed, College of Forestry, Jiangxi Agricultural University, Nanchang, China
[7] Department of Global Development and Department of Ecology and Evolutionary Biology, Cornell University, Ithaca, USA

*Author for correspondence: E-mail: liuxueyan@tju.edu.cn

Table s1. Fractional contributions of different N species to total N of non-leguminous terrestrial plants based on different methods. Lat.: Latitude. MAT: mean annual temperature. ON: organic N. EIN: extractable inorganic N. EON: extractable organic N. Gly: glycine. Arg: arginine. Glu: glutamate. Tri: trialanine. Ser: serine. Asp: aspartate. Mean ± SD are shown. -: not available. The literature (Ref.) of data sources is listed in Supplementary Text 4.

**Pattern**	**Ecosystem**	**Lat. (°)**	**MAT (℃)**	**Method**	**Fractional contributions (%)**			**Ref.**
					**Organic N**	**NH_4^+**	**NO_3^-**	
MAT < 5℃, ON-dominated	Polar tundra	68.5	-10	Lab-growing and ^{14}C labeling for 10 minutes	60 (Gly, Asp, Glu)	-	-	1
	Polar boreal forest	64.3	1.8	Field ^{15}N labeling for 6 hours	65.7 (Arg)	-	-	2
	Polar boreal forest	63.3	-0.5	Lab-growing and ^{15}N labeling for 2 hours	66.0 (Arg)	30.5	3.5	3
	Polar tundra	68.6	-8.8	Natural ^{15}N analyses	42.9±19.8 (EON)	23.2±11.0	33.5±9.7	4
MAT < 5℃, EIN-dominated, especially NH_4^+	Polar boreal	64.7	1.0	Field ^{15}N labeling at 10 cm for 4 hours	15.3±12.4 (Gly)	60.8±14.1	23.8±14.1	5
	Subtropical alpine	31.6	2.3	Field ^{15}N labeling at 15 cm for 6 hours	16.1 (Gly)	56.6	27.3	6
	Polar tundra	68.5	-10	Field ^{15}N labeling at 3, 8 cm for 1 week	31.3±14.0 (Arg)	43.9±19.7	24.3±27.5	7
	Subtropical alpine	37.6	-1.7	Field ^{15}N labeling	23.7±16.6 (Gly)	41.3±24.2	35.0±22.0	8
MAT < 5℃, EIN-dominated, especially NO_3^-	Temperate forest	41.9	4.6	Field ^{15}N labeling at 10 cm for 2 days	22.8±13.3 (Gly)	14.2±12.0	63.0±23.7	9
	Temperate forest	41.9	4.6	Natural ^{15}N analyses	19.6±4.3 (EON)	23.7±11.8	56.6±14.6	9
	Temperate grassland	43.5	0.3	Field ^{15}N labeling at 10 cm for 1 day	5.7±0.9 (Gly)	46.1±24.6	48.2±23.9	10
	Temperate forest	46.4	2.7	Natural ^{15}N analyses	28.7±3.0 (EON)	23.9±4.6	47.4±4.0	11
	Subtropical subalpine	29.5	3.8	Natural ^{15}N analyses	12.8±5.7 (EON)	43.3±15.0	44.7±20.7	12
	Temperate forest	46.4	2.7	Field ^{15}N labeling at 0-20 cm	32.4±18.5 (Gly)	24.5±10.2	43.0±9.4	11
	Polar tundra	68.4	0.5	Field ^{15}N labeling for 26 days	24.4 (Gly)	33.8	41.7	13
	Polar desert	75.2	-16	Lab-growing and ^{15}N labeling for 3 weeks	24.6 (Gly)	34.2	41.3	14
	Subtropical subalpine	33.5	0.6	Field ^{15}N labeling at 2.5 cm for 2 hours	27.5±12.7 (Gly)	34.3±9.8	38.2±11.3	15

MAT > 12℃, EIN-dominated, with quite low contributions from ON	Subtropical forest	26.7	17.9	Field ^{15}N labeling at 15 cm for 2 hours	5.3±2.1 (Gly)	81.2±2.0	13.6±2.8	16
	Tropical forest	-4.1	14.7	Field ^{15}N labeling for 5 days	8.7±8.4 (Gly)	55.0±18.6	36.4±11.7	17
	Subtropical forest	-31.3	21.0	Natural ^{15}N analyses	5.0±8.0 (EON)	17.0±12.0	77.5±13.5	18
	Tropical forest	20.7	28.0	Natural ^{15}N analyses	4.9±4.9 (EON)	35.5±48.4	60.7±46.8	19
	Tropical forest	8.8	20.0	Field ^{15}N labeling for 2 days	12.1 (Gly)	31.0	57.0	20
	Subtropical forest	35.8	12.6	Natural ^{15}N analyses	13.9±6.7 (EON)	33.6±18.0	52.6±23.2	21
MAT > 12℃, EIN-dominated, but with high contributions from ON	Tropical forest	8.2	20.0	Field ^{15}N labeling at 5 cm for 2 hours	39.0 (Gly)	29.0	32.0	22
	Subtropical desert	36.8	12.7	Lab-growing and ^{15}N labeling for 4 hours	33.6 (Gly)	34.9	31.4	23
	Subtropical grassland	37.8	12.9	Field ^{15}N labeling for 2 hours	23.9±22.4 (Gly)	73.7±22.3	2.5±1.2	24
	Tropical forest	22.0	21.8	Lab-growing and ^{15}N labeling for 2 hours	22.9±6.9 (Gly)	64.2±10.4	12.9±6.7	25
	Tropical grassland	-1.9	20.0	Lab-growing and ^{15}N labeling for 1 day	19.6 (Urea)	41.6	38.8	26
10℃ > MAT > 6℃, ON-dominated	Temperate forest	44.2	9.4	Field ^{15}N labeling at 10 cm	66.7 (Gly)	25.0	8.3	27
	Temperate forest	41.8	8.6	Lab-growing and ^{15}N labeling for 1 hour	64.5 (Gly, Arg, Glu, Ser)	32.1	3.4	28
	Subtropical subalpine	31.7	8.9	Natural ^{15}N analyses	35.6±6.9 (EON)	34.9±9.8	30.1±14.5	29
10℃ > MAT > 6℃, EIN-dominated, especially NH_4^+	Temperate grassland	53.2	8.1	Field ^{15}N labeling at 0-8 cm for 2.5 hours	34.8 (Gly, Tri)	49.7	15.5	30
	Temperate forest	51.1	9.0	Field ^{15}N labeling at 0 cm for 7 years	31.2 (Urea)	39.1	29.7	31
	Subtropical forest	40.0	6.3	Lab-growing and ^{15}N labeling for 2 hours	19.6±7.4 (Gly)	75.9±8.4	4.5±1.8	19
	Temperate forest	50.6	7.9	Lab-growing and ^{15}N labeling for 1 day	15.6 (Gly, protein, tannin)	48.8	35.6	32
	Temperate desert	45.5	7.3	Field ^{15}N labeling at 0-15 cm for 1 day	20.2 (Gly)	42.5	37.2	33
10℃ > MAT > 6℃, EIN-dominated, especially NO_3^-	Temperate grassland	48.8	9.3	Field ^{15}N labeling at 0 cm for 1 day	0.3 (Gly)	2.2	97.5	34
	Temperate grassland	47.6	7.4	Field ^{15}N labeling at 0-12 cm for 2 days	26.2±6.8 (Gly)	32.8±9.0	46.1±10.9	35
	Subtropical forest	39.3	7.3	Lab-growing and ^{15}N labeling at 0 cm for 5 days	24.4 (Gly)	36.9	38.7	36

Table s2. Effects of increasing N deposition, temperature, and precipitation on fractional contributions of different N species to non-leguminous terrestrial plants. EON: extractable organic N. Gly: glycine. Arg: arginine. Lat.: Latitude. MAP: mean annual precipitation. -: not available. Mean ± SD are shown. The literature (Ref.) of data sources is listed in Supplementary Text 5.

Factor	Ref.	Ecosystem	Method	Treatment	Fractional contributions (%)		
					Organic N	NH_4^+	NO_3^-
Simulated N deposition (kg-N/ha/yr)	1	Temperate forest	Root ^{15}N-tracer uptake under N additions for 1 hour	0	22.0±11.4 (Gly)	70.6±11.5	7.4±3.8
				50	16.2±0.9 (Gly)	77.8±4.6	6.0±4.7
	2	Temperate grassland	Whole-plant ^{15}N-tracer uptake under N additions at 10 cm for 1 day	0	4.2±1.9 (Gly)	58.8±13.5	37.0±12.9
				20	2.5±1.4 (Gly)	52.2±29.6	45.3±28.7
				200	1.5±0.7 (Gly)	74.8±8.2	23.7±8.1
				500	4.1±1.2 (Gly)	39.7±12.3	56.3±11.1
	3	Tropical forest	Natural ^{15}N analyses of plants under N additions	0	-	48.6±16.5	51.4±16.5
				150	-	22.4±12.4	77.6±12.4
Temperature gradients (℃)	4	Temperate forest	Root ^{15}N-tracer uptake of field plants under increased temperature for 1 day	10	38.9 (Arg)	53.4	7.7
				16	25.8 (Arg)	52.1	22.1
				20	25.9 (Arg)	38.7	35.5
Artificial precipitation reduction (%)	1	Temperate forest	Root ^{15}N-tracer uptake of field plants for 1 day	0	22.0 (Gly)	70.6±11.5	7.4±3.8
				30	22.2 (Gly)	69.9±1.3	7.9±1.9
MAP gradients (mm)	5	Tropical forest	Natural ^{15}N analyses of plants under different MAP	2200	5.0 (EON)	15.0	80.0
				2450	5.0 (EON)	17.0	78.0
				2750	5.0 (EON)	15.0	80.0
				3350	2.0 (EON)	5.0	93.0
				4050	2.0 (EON)	93.0	5.0
				5050	2.0 (EON)	93.0	5.0

Table s3. Effects (shown as *p* values) of MAT, MAP, and life form on $\delta^{15}N_{plant}$ variations of no mycorrhizae (NM), arbuscular mycorrhizae (AM), ectomycorrhizal (ECM), and ericoid mycorrhizal (ERM) plants. The *p* value lower than 0.05 indicates a significant effect. r^2 is the explained variance of the model. Analyses were conducted by the General Linear Model.

$\delta^{15}N_{plant}$	NM (r^2 = 0.238)	AM (r^2 = 0.225)	ECM (r^2 = 0.127)	ERM (r^2 = 0.128)
MAT	0.00	0.00	0.00	0.00
MAP	0.00	0.00	0.17	0.00
Life form	0.00	0.00	0.00	0.00

Table s4. Relationships between isotope effects (Δ_m) of plant N acquisition and MAT variations. Each relationship was formulated by using the fitting formula between $\delta^{15}N_{plant}$ and MAT of the NM plants (Figs. s5a, e, i) minus that of the AM (Figs. s5b, f, j), ECM (Figs. s5c, g, k), or ERM (Figs. s5d, h, l) plants of the same life form. *n* represents sizes of sample replicates (Fig. s4).

**Life form**	**Mycorrhizal type**	**Relationship**	**MAT range (°C)**	**Δ_m range (‰)**	***n***
Herb	AM	Δ_m = -0.05 × MAT + 2.24	-18.7 – 28.5	0.8 – 3.2	12558
Herb	ECM	Δ_m = -0.01 × MAT + 2.77	-9.8 – 26.7	2.5 – 2.9	413
Herb	ERM	Δ_m = -0.46 × MAT + 2.62	-10.6 – 10.6	-2.3 – 7.5	300
Shrub	AM	Δ_m = -0.06 × MAT + 4.26	-10.6 – 28.4	2.6 – 4.9	9437
Shrub	ECM	Δ_m = -0.09 × MAT + 5.23	-18.7 – 27.2	2.8 – 6.9	812
Shrub	ERM	Δ_m = 0.09 × MAT + 6.10	-18.7 – 26.0	4.4 – 8.4	3160
Tree	AM	Δ_m = -0.04 × MAT + 4.46	-4.3 – 28.4	3.3 – 4.6	14193
Tree	ECM	Δ_m = 0.07 × MAT + 3.84	-16.3 – 28.2	2.7 – 5.8	7608
Tree	ERM	Δ_m = 0.10 × MAT + 3.86	7.9 – 26.0	4.7 – 6.5	317

Table s5. Effects of major variables on variations of $\delta^{15}N_{soil\text{-}NO3\text{-}}$, $\delta^{15}N_{soil\text{-}NH4+}$, $\delta^{15}N_{soil\text{-}EON}$ in terrestrial ecosystems. Analyses were conducted by multiple linear regression. r^2 is the explained variance of the model. The p value lower than 0.05 indicates a significant effect. SC denotes the standardized coefficient (positive and negative values show positive and negative effects, respectively).

**Variables**	**$\delta^{15}N_{soil\text{-}NO3\text{-}}$ (r^2 = 0.149)**		**$\delta^{15}N_{soil\text{-}NH4+}$ (r^2 = 0.332)**		**$\delta^{15}N_{soil\text{-}EON}$ (r^2 = 0.737)**	
	p value	SC	*p* value	SC	*p* value	SC
MAT	0.032	0.427	0.000	0.673	0.020	0.982
MAP	0.463	-0.152	0.329	-0.160	0.785	-0.059
Soil density	0.530	0.101	0.981	0.003	0.063	-0.379
Soil clay	0.116	-0.247	0.057	-0.258	0.538	-0.073
Soil pH	0.123	0.256	0.885	-0.019	0.385	-0.630
Soil organic C	0.895	-0.021	0.579	-0.067	0.058	-0.882
N deposition	0.677	0.074	0.823	0.032	0.653	0.130

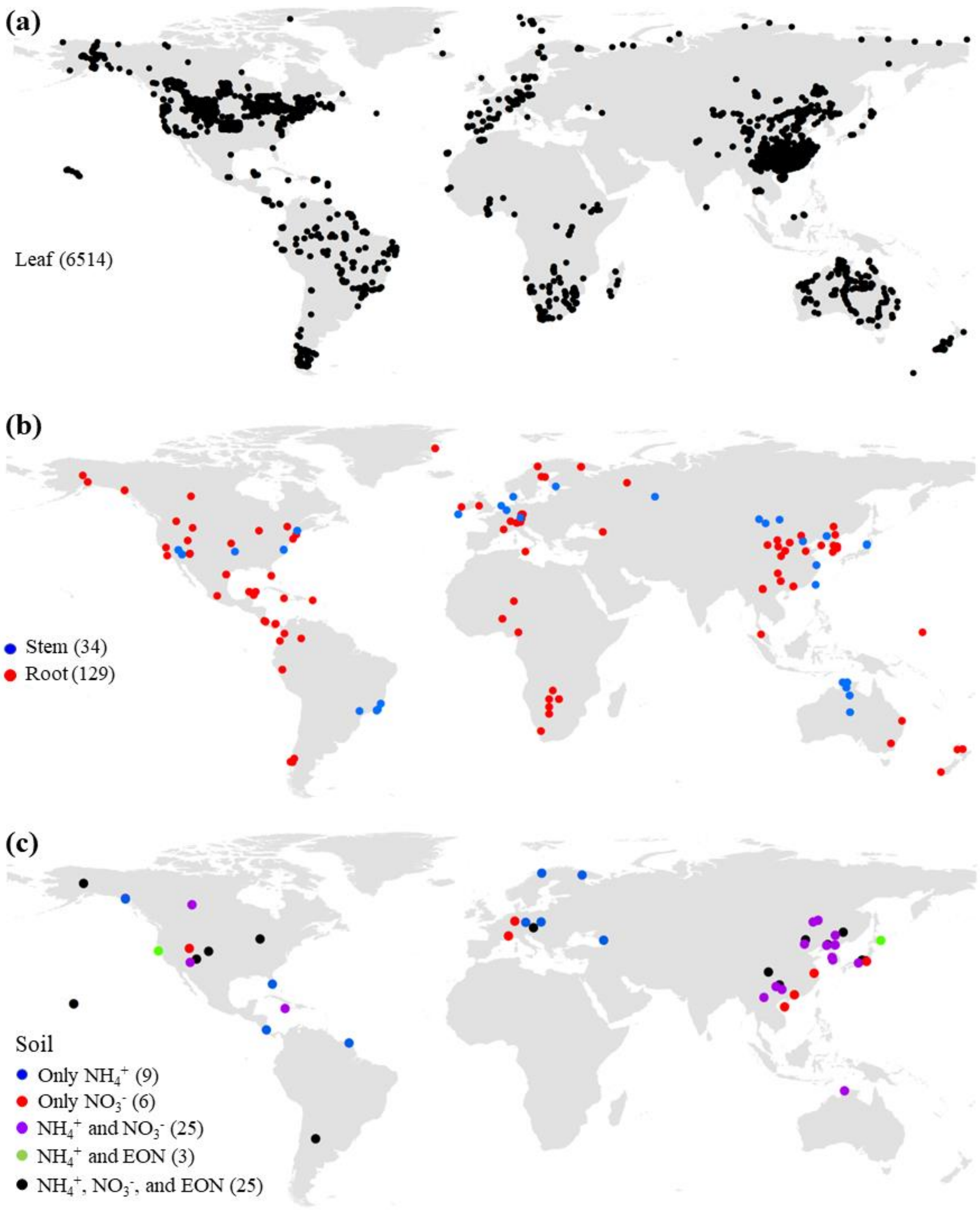

Fig. s1. $\delta^{15}N$ observation sites of leaves (a), stems and roots (b), and soils (c) in terrestrial ecosystems. NO_3^-: nitrate, NH_4^+: ammonium, EON: extractable organic N. Numbers in brackets are site replicates. Leaf $\delta^{15}N$ observations were available at 104 out of 129 sites with root $\delta^{15}N$ observations in panel b and have been included in panel a. Maps were created by using ArcGIS version 10.8 (Esri Inc., USA). The base map was download from https://hub.arcgis.com/datasets/esri::world-countries-generalized.

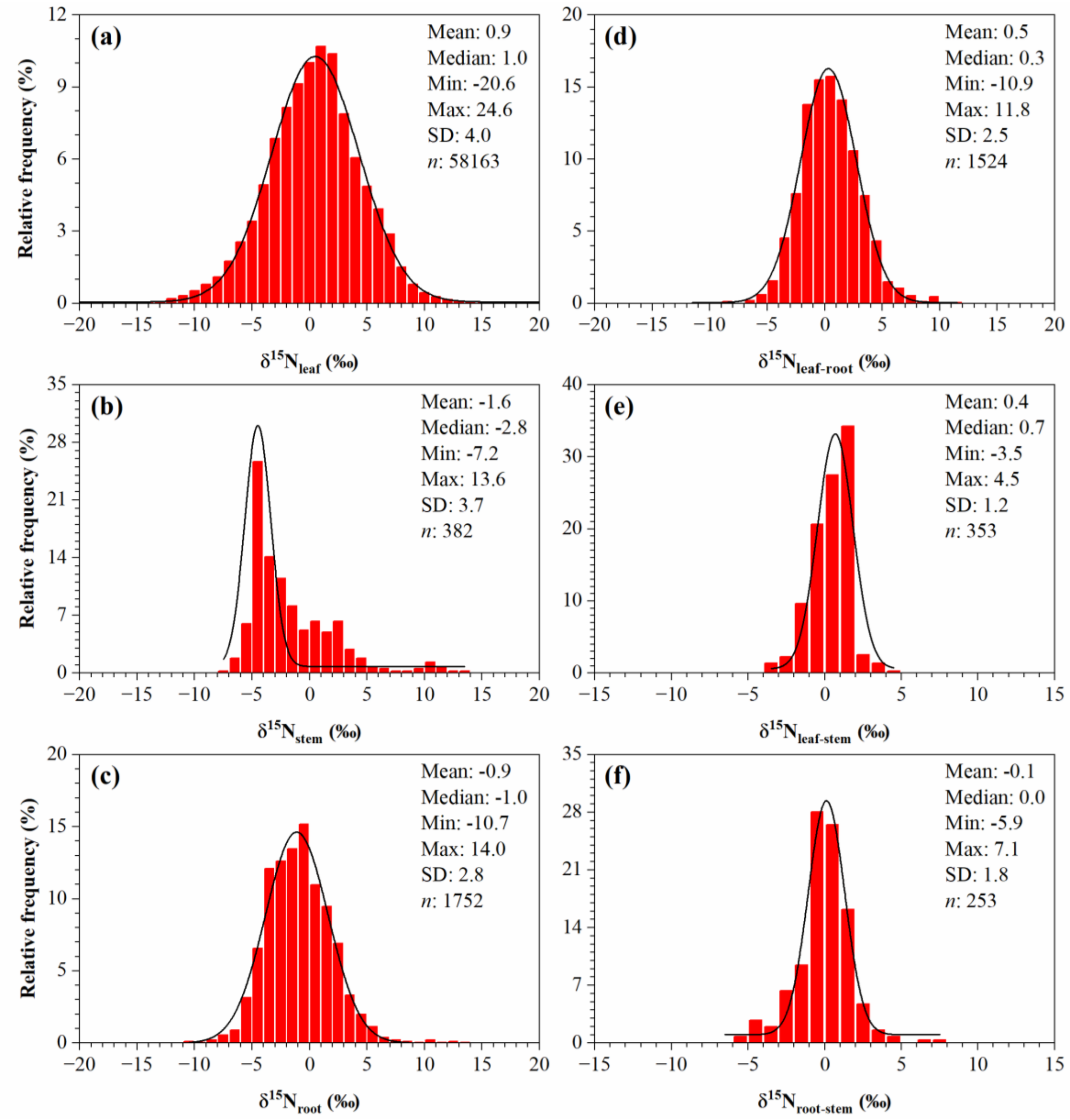

Fig. s2. Relative frequency histograms of terrestrial $\delta^{15}N_{leaf}$ (a), $\delta^{15}N_{stem}$ (b), $\delta^{15}N_{root}$ (c), $\delta^{15}N_{leaf\text{-}root}$ (d), $\delta^{15}N_{leaf\text{-}stem}$ (e), and $\delta^{15}N_{root\text{-}stem}$ (f) values. *n*: sample replicates. The $\delta^{15}N_{leaf\text{-}root}$ and $\delta^{15}N_{leaf\text{-}stem}$ value was calculated by using leaf $\delta^{15}N$ minus root and stem $\delta^{15}N$ of the same plant individual(s). The $\delta^{15}N_{root\text{-}stem}$ value was calculated by using root $\delta^{15}N$ minus stem $\delta^{15}N$ of the same plant individual(s). The solid curve is a Gaussian distribution fitted to the frequency data.

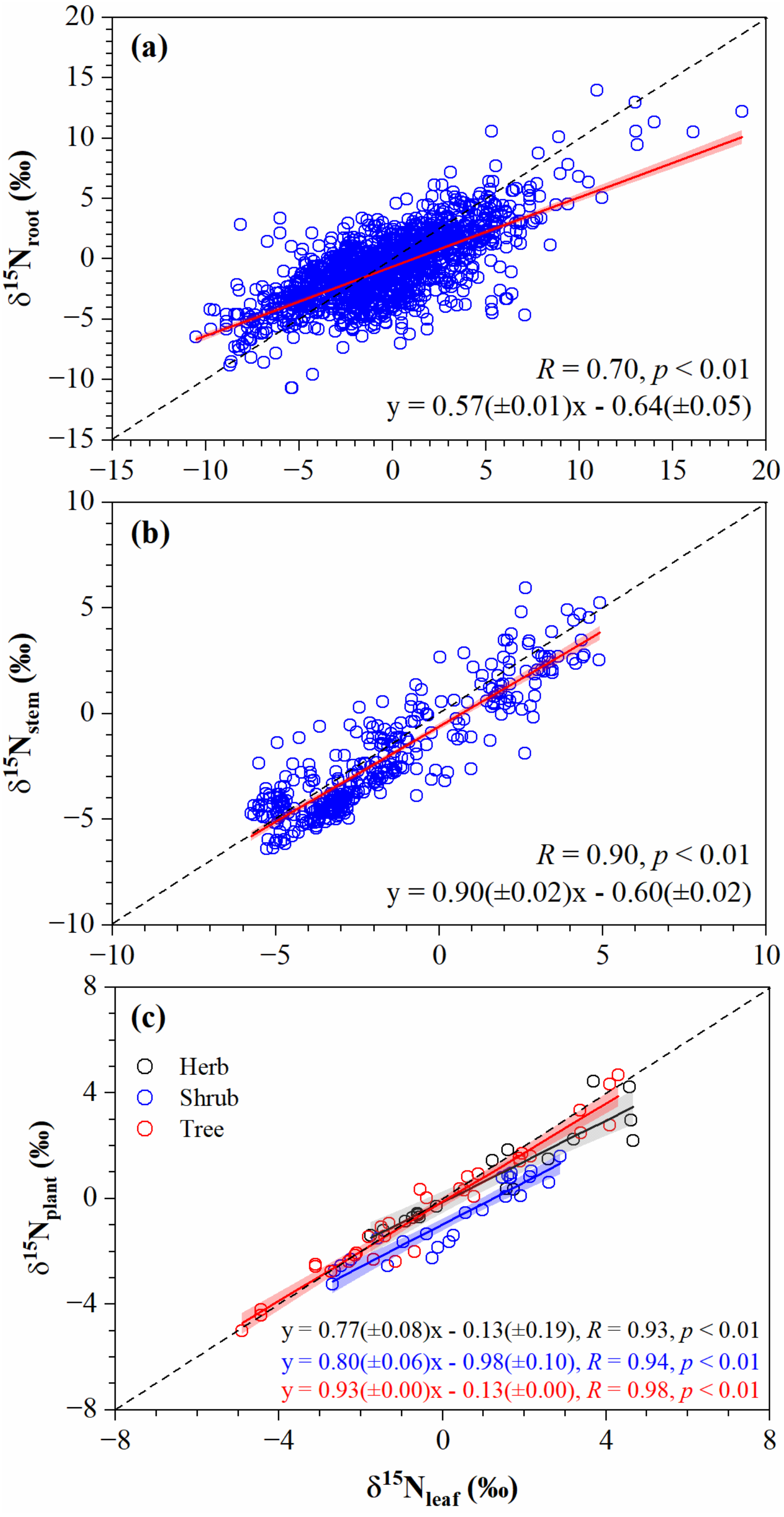

Fig. s3. Correlations between $\delta^{15}N_{leaf}$ and $\delta^{15}N_{stem}$ or $\delta^{15}N_{root}$ (a-c), and between $\delta^{15}N_{plant}$ and $\delta^{15}N_{leaf}$ (d) observed simultaneously on the same plant individuals (including herbs, shrubs, and trees). The $\delta^{15}N_{plant}$ calculation was detailed in Methods (Eqs. 1, 2). $\delta^{15}N$ and N concentration data were collected from the source publications listed in Supplementary Text 2. The regression was analyzed by fitting linear effects with 95% confidence intervals.

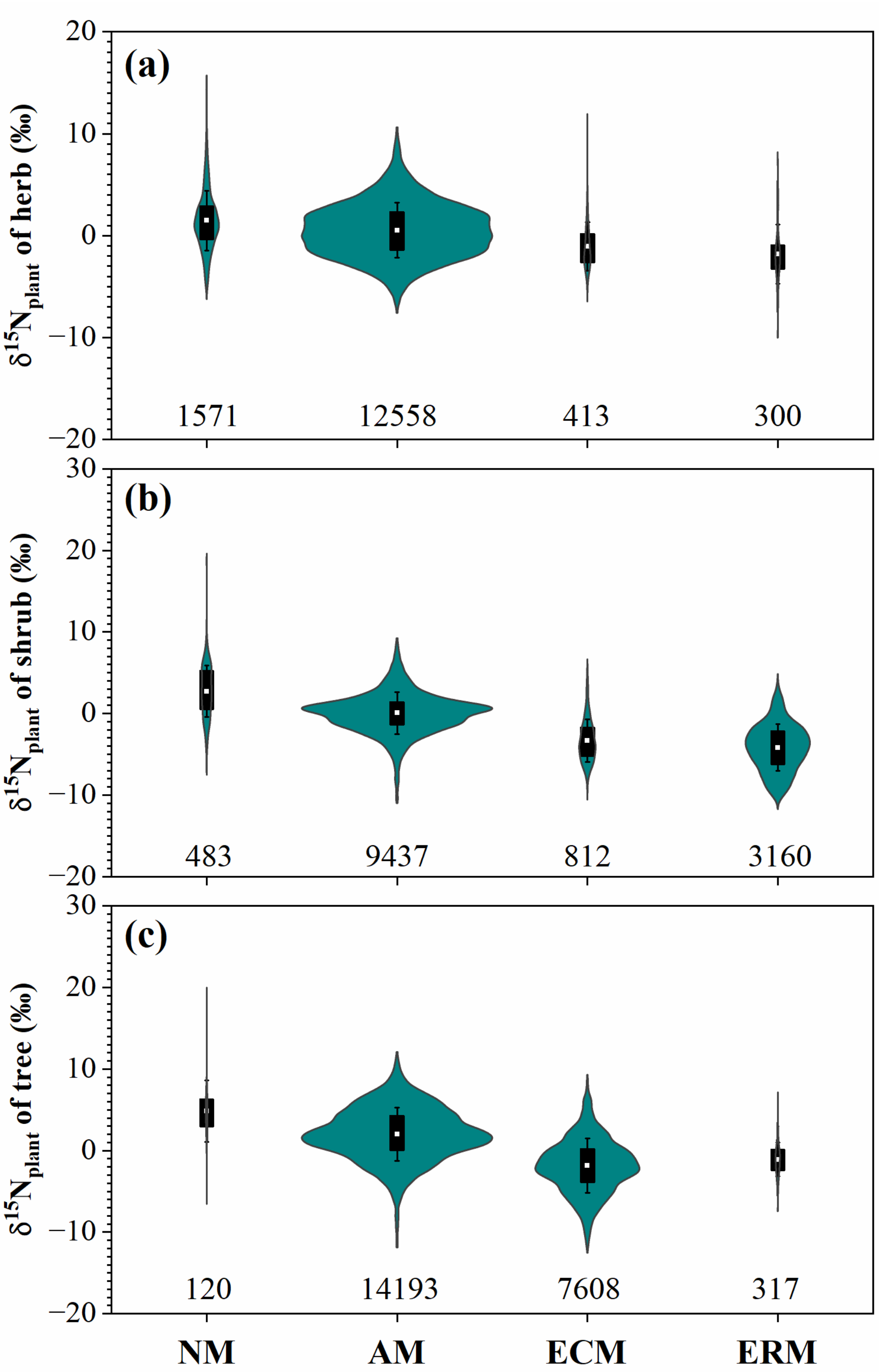

Fig. s4. $\delta^{15}N_{plant}$ values of herb (a), shrub (b), and tree (c) plants associated with NM, AM, ECM, and ERM. Calculations of $\delta^{15}N_{plant}$ values are detailed in Methods. The width of the density plot (in green) represents the frequency distribution of $\delta^{15}N_{plant}$ values. The black-filled box encompasses the 25th–75th percentiles, whiskers are the SD values, and the white-filled circle in each box marks the mean values. Numbers under the boxes are sizes of sample replicates.

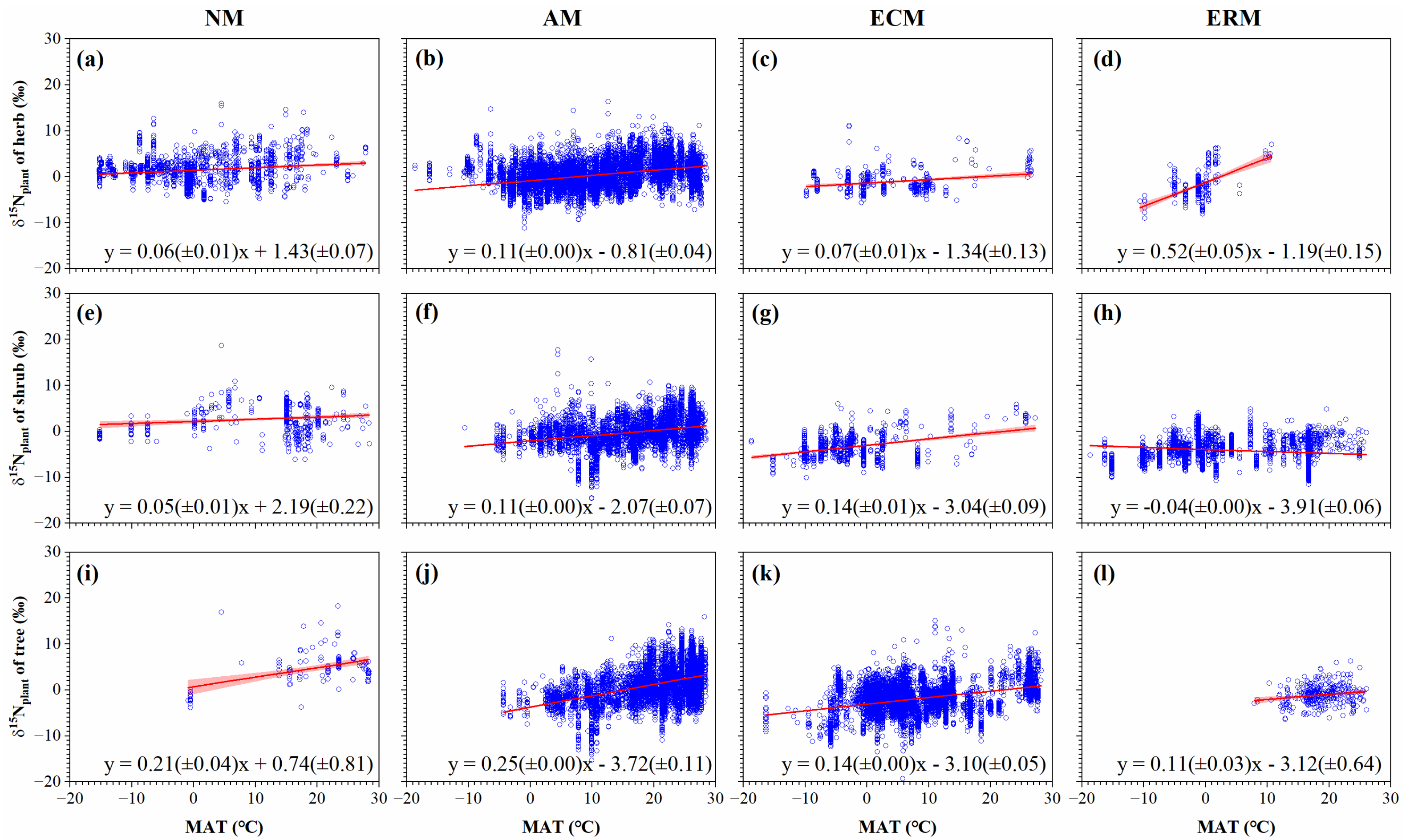

Fig. s5. Variations of $\delta^{15}N_{plant}$ values of herb (a-d), shrub (e-h), and tree (i-l) plants associated with NM (a,e,i), AM (b,f,j), ECM (e,g,k), and ERM (d,h,l) with MAT. Calculations of $\delta^{15}N_{plant}$ values are detailed in Methods. The regression was analyzed by fitting linear effects with 95% confidence intervals.

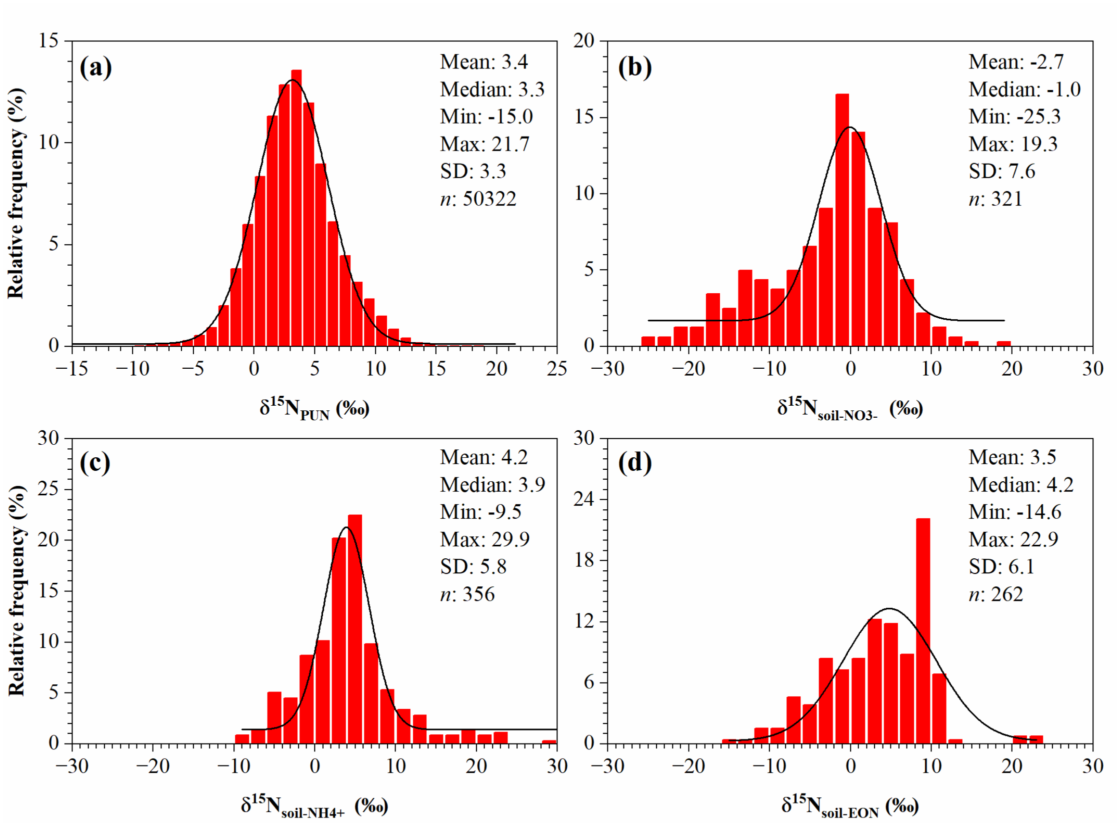

Fig. s6. Relative frequency histograms of terrestrial $\delta^{15}N_{PUN}$ (a), $\delta^{15}N_{soil\text{-}NO3\text{-}}$ (b), $\delta^{15}N_{soil\text{-}NH4+}$ (c), and $\delta^{15}N_{soil\text{-}EON}$ (d) values. *n*: sample replicates. Calculations of $\delta^{15}N_{PUN}$ values are detailed in Methods. The solid curve is a Gaussian distribution fitted to the frequency data.

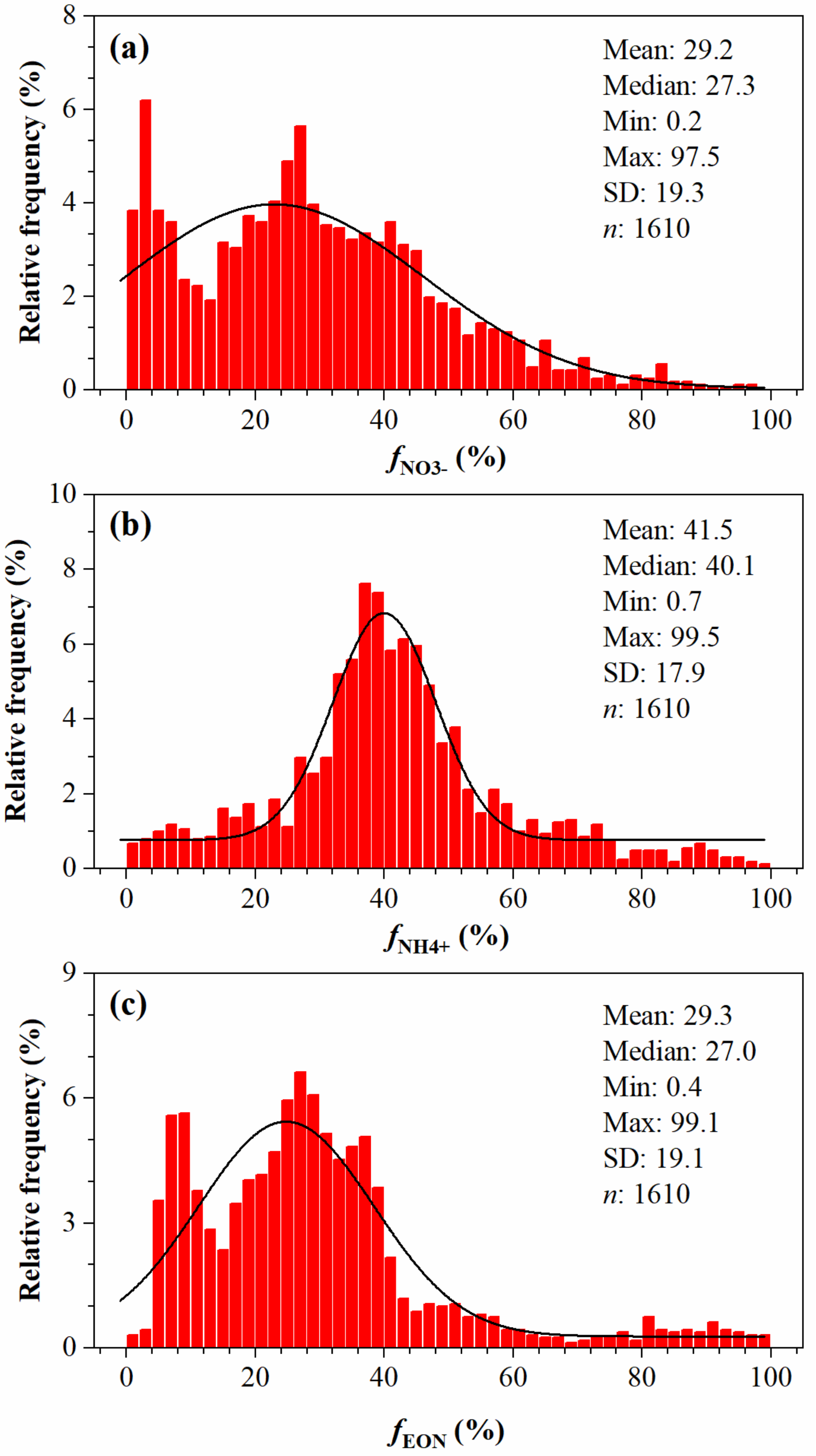

Fig. s7. Relative frequency histograms of f_{NO3-} (a), f_{NH4+} (b), and f_{EON} (c) values of terrestrial plants. *n*: sample replicates. Calculations of *f* values are detailed in Methods. The solid curve is a Gaussian distribution fitted to the frequency data.

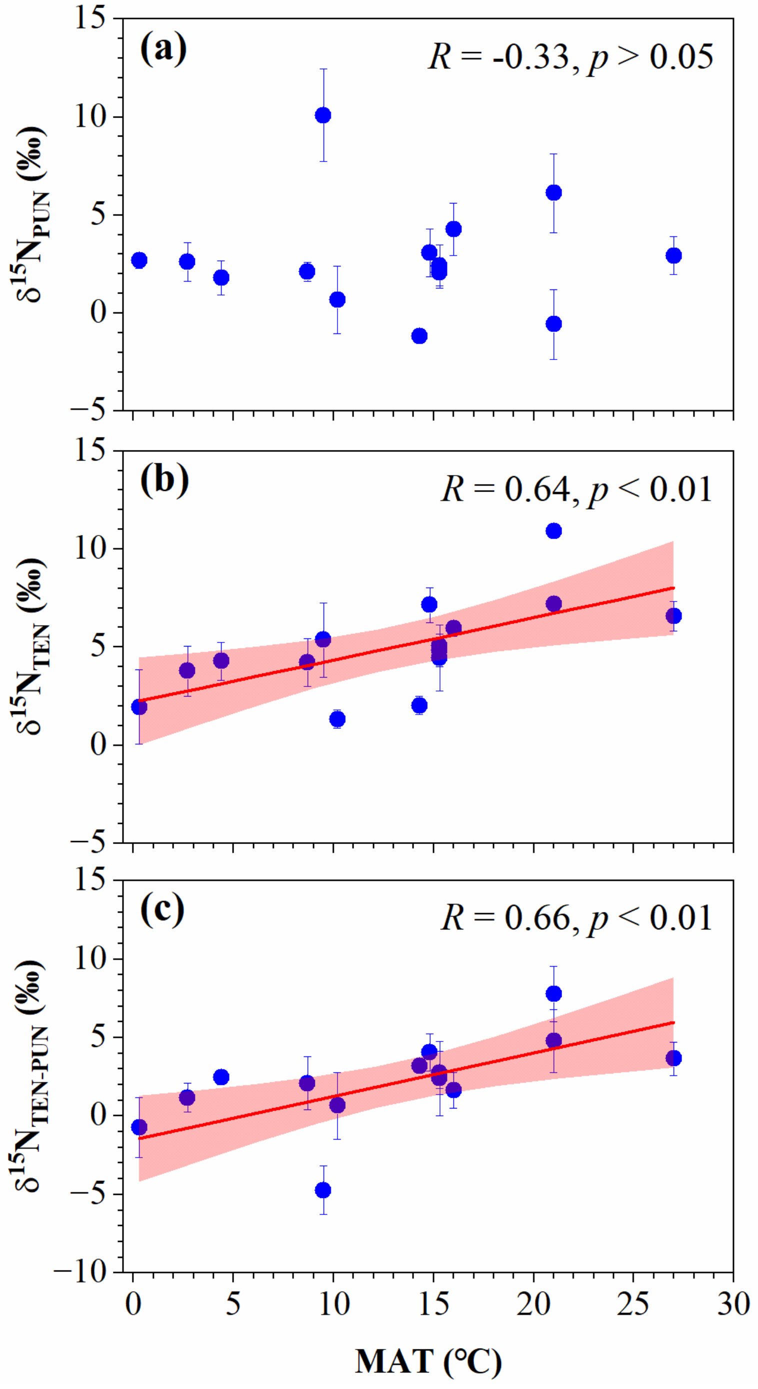

Fig. s8. Variations of $\delta^{15}N_{PUN}$ (a), $\delta^{15}N_{TEN}$ (b), and $\delta^{15}N_{TEN\text{-}PUN}$ (c) in soils with MAT in terrestrial ecosystems. Mean and SD values based on sample replicates at each site (*n* = 2–16) are shown. For each site, $\delta^{15}N_{leaf}$, $\delta^{15}N_{soil\text{-}NO3\text{-}}$, $\delta^{15}N_{soil\text{-}NH4+}$, $\delta^{15}N_{soil\text{-}EON}$, soil NO_3^-, NH_4^+, EON, TEN concentrations were simultaneously observed. These sites have been included in global patterns (Figs. s1-2). PUN: plant-used N in soil. Calculations of $\delta^{15}N_{PUN}$ values are detailed in Methods. $\delta^{15}N_{TEN} = \delta^{15}N_{soil\text{-}NO3\text{-}} \times NO_3^-/TEN + \delta^{15}N_{soil\text{-}NH4+} \times NH_4^+/TEN + \delta^{15}N_{soil\text{-}EON} \times EON/TEN$. The $\delta^{15}N_{TEN\text{-}PUN}$ value was calculated by using $\delta^{15}N_{TEN}$ minus $\delta^{15}N_{PUN}$ at each site. The regression was analyzed by fitting linear effects with 95% confidence intervals.

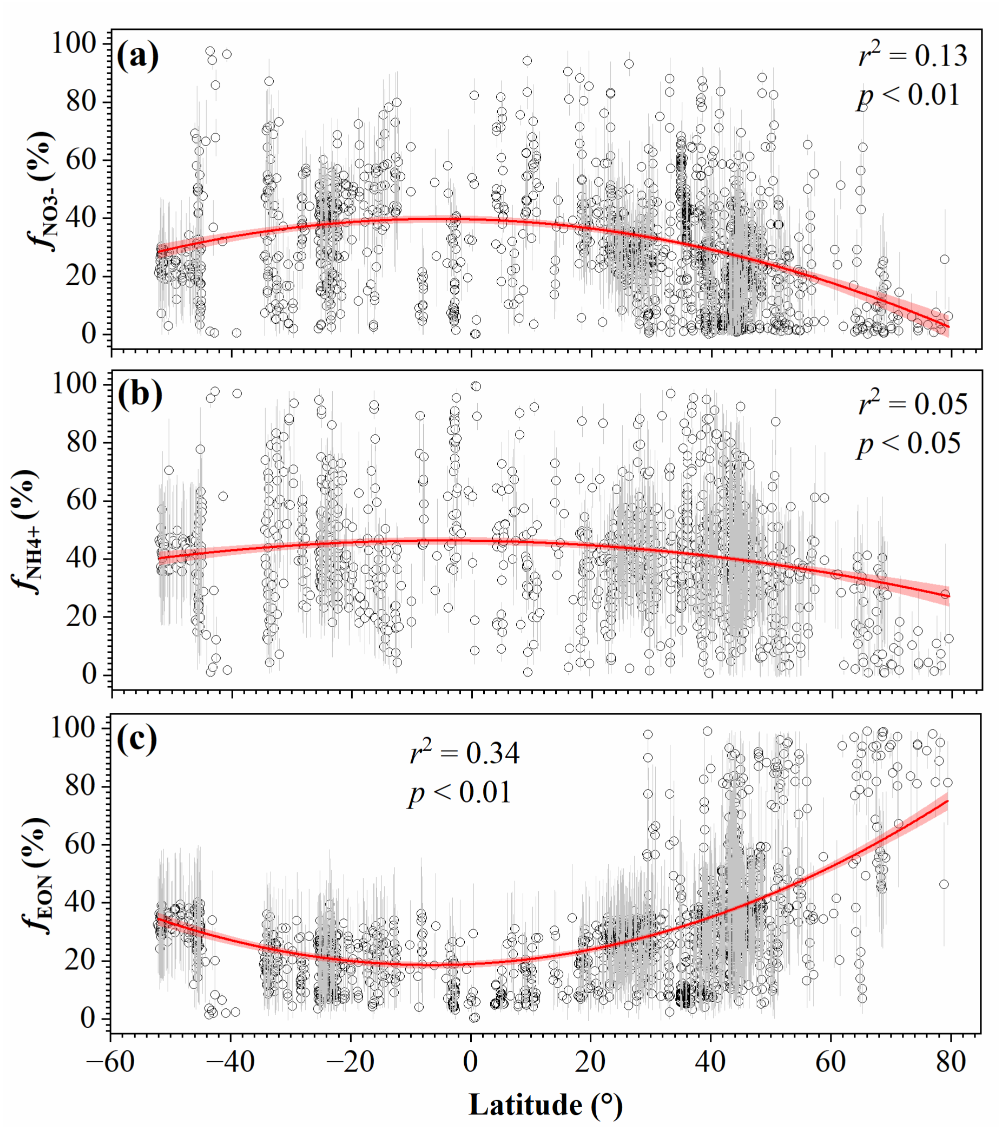

Fig. s9. Variations of f_{NO3-} (a), f_{NH4+} (b), and f_{EON} (c) with latitude in terrestrial ecosystems. Calculations of f values are detailed in Methods. The 0.1° (latitude) × 0.1° (longitude) grid-based mean ± SD values (n = 3–1028 for each grid) are shown. Analyses were conducted by regressive analysis.

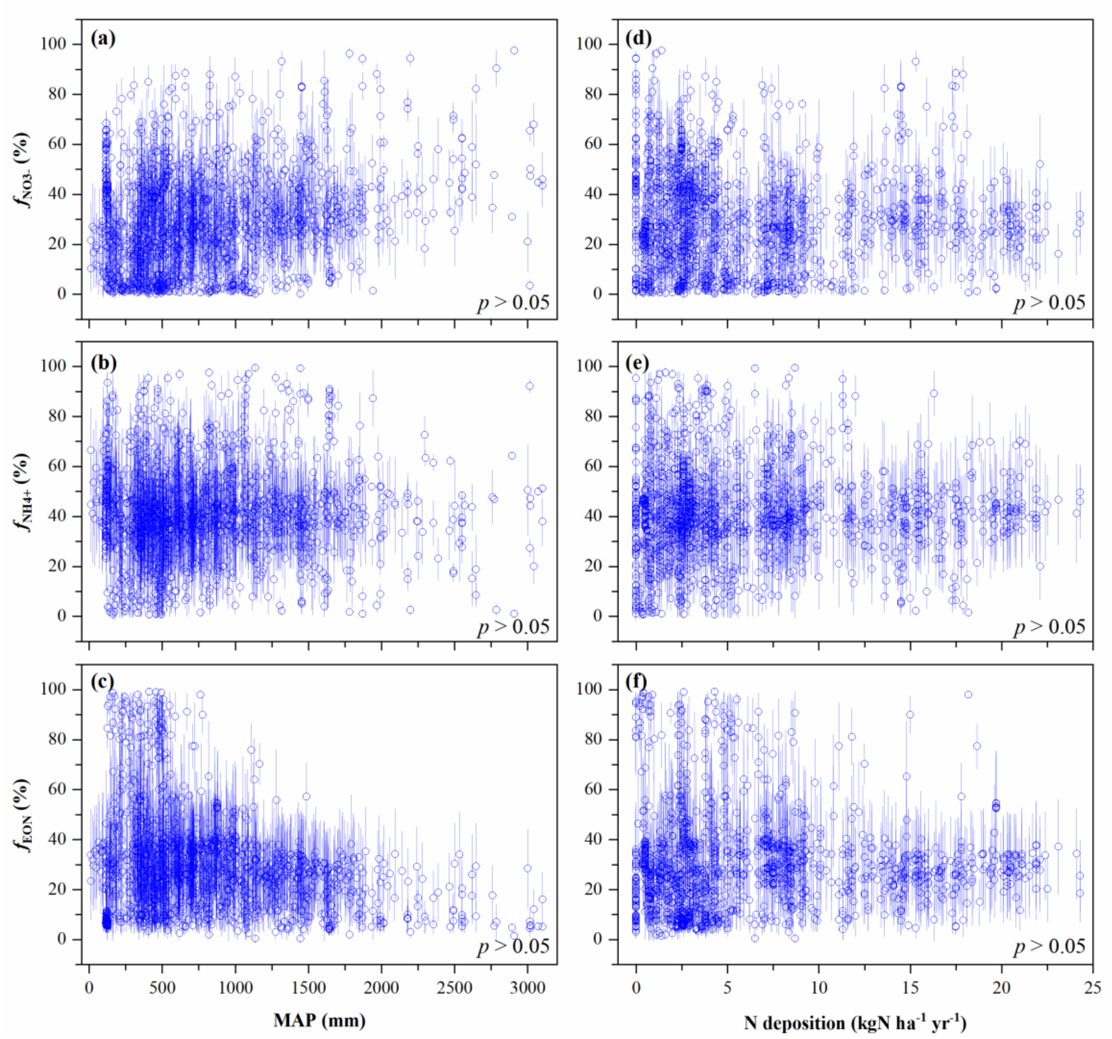

Fig. s10. Variations of f_{NO3-} (a, d), f_{NH4+} (b, e), and f_{EON} (c, f) with MAP (a-c) and N deposition (d-f) in terrestrial ecosystems. Calculations of f values are detailed in Methods. The 0.1° (latitude) × 0.1° (longitude) grid-based mean ± SD values (n = 3–1028 for each grid) are shown. Analyses were conducted by regressive analysis. The p value lower than 0.05 indicates a significant effect.

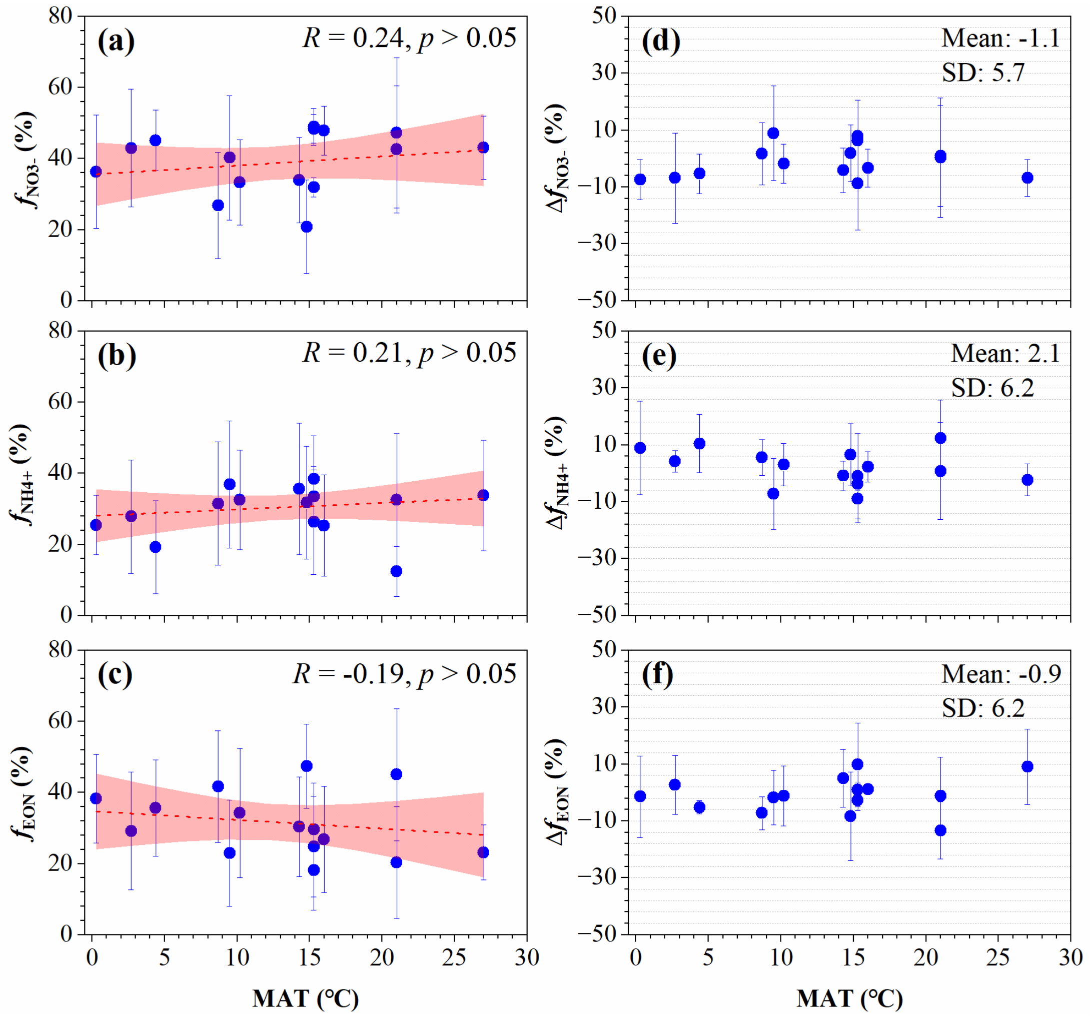

Fig. s11. Variations of plant f_{NO3-} (a), f_{NH4+} (b), f_{EON} (c), Δf_{NO3-} (d), Δf_{NH4+} (e), and Δf_{EON} (f) with MAT at study sites with simultaneous observations on $\delta^{15}N_{leaf}$, $\delta^{15}N_{soil\text{-}NO3\text{-}}$, $\delta^{15}N_{soil\text{-}NH4+}$, $\delta^{15}N_{soil\text{-}EON}$, soil NO_3^-, NH_4^+, EON, and TEN concentrations (Fig. s8). Site-based mean and SD values are shown. Calculations of *f* values are detailed in Methods. Each Δf value was calculated by using the *f* value of the global database in Fig. 4 minus the corresponding *f* value of the same MAT in panels a-c.

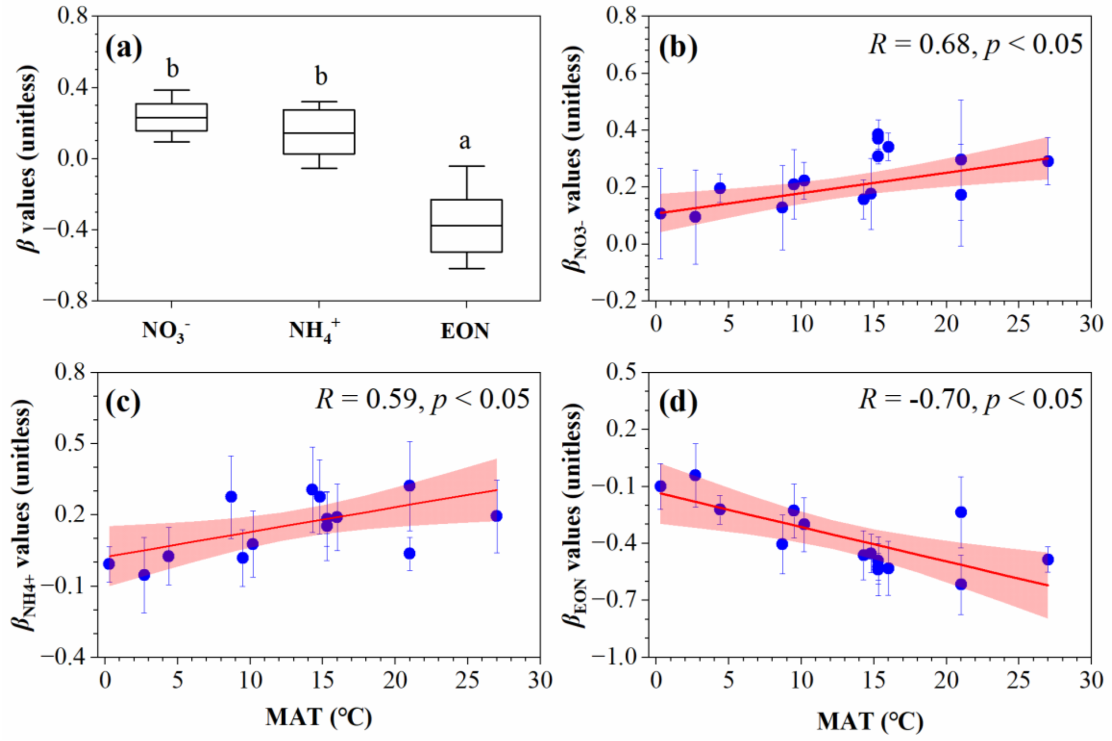

Fig. s12. Plant β_{NO3-}, β_{NH4+}, and β_{EON} values (a) and their variations with MAT (b-d) at study sites with simultaneous observations on $\delta^{15}N_{leaf}$, $\delta^{15}N_{soil\text{-}NO3\text{-}}$, $\delta^{15}N_{soil\text{-}NH4+}$, $\delta^{15}N_{soil\text{-}EON}$, soil NO_3^-, NH_4^+, EON, and TEN concentrations (Fig. s8, s11). Calculations of β values are detailed in Methods. Site-based mean and SD values are shown. The regression was analyzed by fitting linear effects with 95% confidence intervals. For panel a, the box encompasses the 25th–75th percentiles, whiskers are the SD values, and the line in each box marks the mean values.

Supplementary Text 1 | Publications of leaf $\delta^{15}N$ observations from January 2018 to August 2022.

Burnett MW, Bobbett AE, Brendel CE, Marshall K, Sperber CV, Paulus EL Vitousek PM. (2022) Foliar $\delta^{15}N$ patterns in legumes and non-N fixers across a climate gradient, Hawaii Island, USA. Oecologia, 198, 229–242.

Brookshire ENJ, Stoy PC, Currey B, Finney B. (2020) The greening of the Northern Great Plains and its biogeochemical precursors. Global Change Biology, 26(10) 5404–5413.

Chen CJ, Jia YF, Chen YZ, Mehmood I, Fang YT, Wang GA. (2018) Nitrogen isotopic composition of plants and soil in an arid mountainous terrain: south slope versus north slope. Biogeosciences, 15, 369–377.

Chen CJ, Liu XY, Wang XW, Hu CC, Xu SQ, Mao R, Bu ZJ, Fang YT, Koba K. (2021) Different leaf carbon, nitrogen, and phosphorus stoichiometry and carbon and nitrogen isotopes among peatland plants in northeastern China. Plant and Soil, 467, 345–357.

Chen Q, Chen J, Andersen MN, Cheng XL. (2022) Elevational shifts in foliar-soil $\delta^{15}N$ in the Hengduan Mountains and different potential mechanisms. Global Change Biology, 28(18) 5480–5491.

Driscoll AW, Kannenberg SA, Ehleringer JR. (2021) Long-term nitrogen isotope dynamics in *Encelia farinosa* reflect plant demographics and climate. New Phytologist, 232, 1226–1237.

Enta A, Hayashi M, Caceres MLL, Fujiyoshi L, Yamanaka T, Oikawa A, Seidel F. (2020) Nitrogen resorption and fractionation during leaf senescence in typical tree species in Japan. Journal of Forest Research, 31(6) 2053–2062.

Ehleringer JR, Sandquist DR. (2018) A tale of ENSO, PDO, and increasing aridity impacts on drought-deciduous shrubs in the Death Valley region. Oecologia, 187, 879–895.

Fujiyoshia L, Sugimotob A, Yamashita Y, Li XY. (2019) Influence of soil N availability on the difference between tree foliage and soil $\delta^{15}N$ from comparison of Mongolia and northern Japan. Ecological Indicators, 101, 1086–1093.

Giesemann P, Eichenberg D, Stöckel M, Seifert LF, Gomes SIF, Merckx VSFT, Gebauer G. (2020) Dark septate endophytes and arbuscular mycorrhizal fungi (*Paris*-morphotype) affect the stable isotope composition of 'classically' non-mycorrhizal plants. Functional Ecology, 34, 2453–2466.

Givnish TJ, Shiba ZW. (2022) Leaf NPK stoichiometry, $\delta^{15}N$, and apparent nutrient limitation of co-occurring carnivorous vs. noncarnivorous plants. Ecology, doi: 10.1002/ecy.3825.

Gurmesa GA, Hobbie EA, Zhang SS, Wang A, Zhu FF, Zhu WX, Koba K, Yoh M, Wang CK, Zhang QL, Fang YT. (2022) Natural ^{15}N abundance of ammonium and nitrate in soil profiles: New insights into forest ecosystem nitrogen saturation. Ecosphere, 13(3) e3998.

Hu CC, Liu XY. (2022) Plant nitrogen-use strategies and their responses to the urban

elevation of atmospheric nitrogen deposition in southwestern China. Environmental Pollution, 311, 119969.

Hu CC, Lei YB, Tan YH, Sun XC, Xu H, Liu CQ, Liu XY. (2019) Plant nitrogen and phosphorus utilization under invasive pressure in a montane ecosystem of tropical China. Journal of Ecology, 107, 372–386.

Hu YK, Liu GF, Pan X, Song YB, Dong M, Cornelissen JHC. (2022) Contrasting nitrogen cycling between herbaceous wetland and terrestrial ecosystems inferred from plant and soil nitrogen isotopes across China. Journal of Ecology, 110, 1259–1270.

Hyodo F, Takebayashi Y, Makabe A, Wardle DA, Koba K. (2020) Changes in stable nitrogen isotopes of plants, bulk soil and soil dissolved N during ecosystem retrogression in boreal forest. Ecological Research, 36, 420–429.

Illuminati A, Querejeta JI, Pías B, Escudero A, Matesanz S. (2022) Coordination between water uptake depth and the leaf economic spectrum in a Mediterranean shrubland. Journal of Ecology, 110(8) 1844–1856.

Liu XY, Kobab K, Koyamae LA, Hobbie SE, Weiss MS, Inagaki Y, Shaver GR, Giblin AE, Hobara S, Nadelhoffer KJ, Sommerkorn M, Rastetter EB, Kling GW, Laundre JA, Yano Y, Makabe A, Yano M, Liu CQ. (2018) Nitrate is an important nitrogen source for Arctic tundra plants. Proceedings of the National Academy of Sciences of the United States of America, 115(13) 3398–3403.

Luo WT, Wang XG, Sardans J, Wang ZW, Dijkstra FA, Lü XT, Peñuelas J, Han XG. (2018) Higher capability of C_3 than C_4 plants to use nitrogen inferred from nitrogen stable isotopes along an aridity gradient. Plant and Soil, 428, 93–103.

Makarova MI, Buzin IS, Tiunov AV, Malyshev I, Kadulin MS, Korolev NE. (2019) Nitrogen isotopes in soils and plants of tundra ecosystems in the Khibiny Mountains. Eurasian Soil Science, 52(10) 1195–1206.

Manninen S, Zverev V, Kozlov MV. (2022) Foliar stable isotope ratios of carbon and nitrogen in boreal forest plants exposed to long-term pollution from the nickel-copper smelter at Monchegorsk, Russia. Environmental Science and Pollution Research, 29, 48880–48892.

Martinelli LA, Nardoto GB, Soltangheisi A, Reis CRG, Abdalla-Filho AL, Camarg PB, Domingues TF, Faria D, Figueira AM, Gomes TF, Lins SRM, Mardegan SF, Mariano E, Miatto RC, Moraes R, Moreira MZ, Oliveira RS, Ometto JPHB, Santos FLS, Sena-Souza J, D. Silva ML, Silva JCSS, Vieira SA. (2021) Determining ecosystem functioning in Brazilian biomes through foliar carbon and nitrogen concentrations and stable isotope ratios. Biogeochemistry, 154, 405–423.

Mostaghimi F, Seyedi N, Shafiei AB, Correia O. (2021) How do leaf carbon and nitrogen contents of oak hosts affect the heterotrophic level of *Loranthus europaeus*? Insights from stable isotope ecophysiology assays. Ecological Indicators, 125, 107583.

Niu WL, Chen H, Wu JS. (2021) Soil moisture and soluble salt content dominate changes in foliar $\delta^{13}C$ and $\delta^{15}N$ of desert communities in the Qaidam Basin, Qinghai-Tibetan plateau. Frontiers in Plant Science, 12, 675817.

Pellitier PT, Zak DR, Argiroff WA, Upchurch RA. (2021) Coupled shifts in

ectomycorrhizal communities and plant uptake of organic nitrogen along a soil gradient: An isotopic perspective. Ecosystems, 24, 1976–1990.

Rivero-Villar A, Templer PH, Parra-Tabla V, Campo J. (2018) Differences in nitrogen cycling between tropical dry forests with contrasting precipitation revealed by stable isotopes of nitrogen in plants and soils. Biotropica, 50(6) 859–867.

Seidel F, Lopez CML, Oikawa A, Yamanaka T. (2019) Seasonal nitrogen partitioning in Japanese cedar (*Cryptomeria japonica*, D. Don) tissues. Plant and Soil, 442, 511–529.

Seidel F, Lopez C ML, Celi L, Bonifacio E, Oikawa A, Yamanaka T. (2019) N isotope fractionation in tree Tissues during N reabsorption and remobilization in *fagus crenata* Blume. Forests, 10(4) 330.

Song WC, Zhou YJ. (2021) Linking leaf $\delta^{15}N$ and $\delta^{13}C$ with soil fungal biodiversity, ectomycorrhizal and plant pathogenic abundance in forest ecosystems of China. Catena, 200, 105176.

Tang SB, Liu JF, Gilliam FS, Hietz P, Wang ZH, Lu XK, Zeng FY, Wen DZ, Hou EQ, Lai Y, Fang YT, Tu Y, Xi D, Huang ZQ, Zhang DX, Wang R, Kuang YW. (2022) Drivers of foliar ^{15}N trends in southern China over the last century. Global Change Biology, 28(18) 5441–5452.

Tatsumi C, Hyodo F, Taniguchi T, Shi WY, Koba K, Fukushima K, Du S, Yamanaka N, Templer P, Tateno R. (2021) Arbuscular mycorrhizal community in roots and nitrogen uptake patterns of understory trees beneath ectomycorrhizal and non-ectomycorrhizal overstory Trees. Frontiers in Plant Science, 11, 583585.

Wang X, Jiang Y, Ren HY, Yu FH, Li MH. (2019) Leaf and soil $\delta^{15}N$ patterns along elevational gradients at both treelines and shrublines in three different climate zones. Forests, 10, 557.

Yu Q, Duan L, Yu LF, Chen X, Si GY, Ke PP, Ye ZX, Mulder J. (2018) Threshold and multiple indicators for nitrogen saturation in subtropical forests. Environmental Pollution, 241, 664–673.

Zeng XH, Ni ZY, Diao HX, Jiang K, Hu C, Shao L, Huang WC. (2021) Root endophytic fungal community and carbon and nitrogen stable isotope patterns differ among *Bletilla* species (Orchidaceae). Journal of Fungi, 7, 69.

Zhang QF, Zhou JC, Li XJ, Yang ZJ, Zheng Y, Wang J, Lin WS, Xie JS, Chen Y, Yang YS. (2019) Are the combined effects of warming and drought on foliar C:N:P:K stoichiometry in a subtropical forest greater than their individual effects? Forest Ecology and Management, 448, 256–266.

Zhou Y, Mushinski RM, Hyodo A, Wu XB, Boutton TW. (2018) Vegetation change alters soil profile $\delta^{15}N$ values at the landscape scale. Soil Biology and Biochemistry, 119, 110–120.

Zhu FF, Dai LM, Hobbie EA, Koba K, Liu XY, Gurmesa GA, Huang SN, Li SL, Li YH, Han SJ, Fang YT. (2019) Uptake patterns of glycine, ammonium, and nitrate differ among four common tree species of northeast China. Frontiers in Plant Science, 10, 799.

Supplementary Text 2 | Publications of $\delta^{15}N$ observations for leaves and roots of the same plant individuals.

An H, Li GQ. (2015) Effects of grazing on carbon and nitrogen in plants and soils in a semiarid desert grassland, China. Journal of Arid Land, 7, 341–349.

Agnelli A, Bol R, Trumbore SE, Dixon L, Cocco S, Corti G. (2014) Carbon and nitrogen in soil and vine roots in harrowed and grass-covered vineyards. Agriculture, Ecosystems and Environment, 193, 70–82.

Ariz I, Cruz C, Neves T, Irigoyen JJ, Garcia-Olaverri C, Nogués S, Aparicio-Tejo PM, Aranjuelo I. (2015) Leaf $\delta^{15}N$ as a physiological indicator of the responsiveness of N_2-fixing alfalfa plants to elevated [CO_2], temperature and low water availability. Frontiers in Plant Science, 6, 574.

Baisden WT, Amundson R, Brenner DL. (2002) A multi-isotope C and N modeling analysis of soil organic matter turnover and transport as a function of soil depth in a California annual grassland soil chronosequence. Global Biogeochemical Cycles, 16, 4.

Boeckx P, Paulino L, Oyarzún C, Cleemput O, Godoy R. (2005) Soil $\delta^{15}N$ patterns in old-growth forests of southern Chile as integrator for N-cycling. Isotopes in Environmental and Health Studies. 41, 249–259.

Bouillet JP, Laclau JP, Goncalves JLM, Moreira MZ, Trivelin PCO, Jourdan C, Silva EV, Piccolo MC, Tsai SM, Galiana A. (2008) Mixed-species plantations of *Acacia mangium* and *Eucalyptus grandis* in Brazil 2: Nitrogen accumulation in the stands and biological N_2 fixation. Forest Ecology and Management, 255, 3918–3930.

Boutton TW, Liao JD. (2010) Changes in soil nitrogen storage and $\delta^{15}N$ with woody plant encroachment in a subtropical savanna parkland landscape. Journal of Geophysical Research: Biogeosciences, 115, G03019.

Brearley FQ. (2013) Nitrogen stable isotopes indicate differences in nitrogen cycling between two contrasting Jamaican montane forests. Plant and Soil, 367, 465–476.

Cameron DD, Bolin JF. (2010) Isotopic evidence of partial mycoheterotrophy in the Gentianaceae: *Bartonia virginica* and *Obolaria virginica* as case studies. American Journal of Botany, 97, 1272–1277.

Cao X, Jia JB, Zhang C, Li H, Liu TX, Jiang XN, Polle A, Peng CH, Luo ZB. (2014) Anatomical, physiological and transcriptional responses of two contrasting poplar genotypes to drought and re-watering. Physiologia Plantarum, 151, 480–494.

Chapman WK, Paul L. (2012) Evidence that northern pioneering pines with tuberculate mycorrhizae are unaffected by varying soil nitrogen levels. Microbial Ecology, 64, 964–972.

Cheng XL, Yang YH, Li M, Dou XL, Zhang QF. (2013) The impact of agricultural land use changes on soil organic carbon dynamics in the Danjiangkou Reservoir area of China. Plant and Soil, 366, 415–424.

Chung IM, Kim JK, Lee JH, An MJ, Lee KJ, Park SK, Kim JU, Kim MJ, Kim SH. (2017) C/N/O/S stable isotopic and chemometric analyses for determining the geographical origin of Panax ginseng cultivated in Korea. Journal of Ginseng Research, 42, 485–495.

Clarkson BR, Schipper LA, Silvester WB. (2009) Nutritional niche separation in coexisting bog species demonstrated by ^{15}N-enriched simulated rainfall. Austral Ecology, 34, 377–385.

Craine JM, Lee WG, Bond WJ, Willlams RJ, Johnson LC. (2005) Environmental constraints on a global relationship among leaf and root traits of grasses. Ecology, 86, 12–19.

Díaz-Álvarez EA, Reyes-García C, de la Barrera E. (2016) A δ^{15}N assessment of nitrogen deposition for the endangered epiphytic orchid *Laelia speciosa* from a city and an oak forest in Mexico. Journal of Plant Research, 129, 863–872.

Dijkstra P, Williamson C, Menyail O, Doucett R, Koch G, Hungate A. (2003) Nitrogen stable roots of plants growing in a forest and a meadow. Isotopes in Environmental and Health Studies, 39, 29–39.

Driscoll AW, Kannenberg SA, Ehleringer JR. (2021) Long-term nitrogen isotope dynamics in *Encelia farinosa* reflect plant demographics and climate. New Phytologist, 232, 1226–1237.

Emmett BA, Kjonaas OJ, Gundersen P, Koopmans C, Tietema A, Sleep D. (1998) Natural abundance of ^{15}N in forests across a nitrogen deposition gradient. Forest Ecology and Management, 101, 9–18.

February EC, Allsopp N, Shabane T, Hattas D. (2011) Coexistence of a C_4 grass and a leaf succulent shrub in an arid ecosystem. The relationship between rooting depth, water and nitrogen. Plant and Soil, 349, 253–260.

Field KJ, Leake JR, Tille S, Allinson KE, Rimington WR, Bidartondo MI, Beerling DJ, Cameron DD. (2015) From mycoheterotrophy to mutualism: mycorrhizal specificity and functioning in *Ophioglossum vulgatum* sporophytes. New Phytologist, 205, 1492–1502.

Franco AC, Duarte HM, Geßler A, de Mattos EA, Nahm M, Rennenberg H, Ribeiro KT, Scarano FR, Luttge U. (2005) In situ measurements of carbon and nitrogen distribution and composition, photochemical efficiency and stable isotope ratios in *Araucaria angustifolia*. Tree, 19, 422–430.

Frank DA, Evans D, Tracy BF. (2004) The role of ammonia volatilization in controlling the natural ^{15}N abundance of a grazed grassland. Biogeochemistry, 68, 169–178.

Gebauer G, Schulze ED. (1991) Carbon and nitrogen isotope ratios in different compartments of a healthy and a declining *Picea abies* forest in the Fichtelgebirge, NE Bavaria. Oecologia, 87, 198–207.

Geβler A, Duarte HM, Franco AC, Luttge U, de Mattos EA, Nahm M, Scarano FR, Zaluar HLT, Rennenberg H. (2005) Ecophysiology of selected tree species in different plant communities at the periphery of the Atlantic Forest of SE-Brazil II. Spatial and ontogenetic dynamics in *Andira legalis*, a deciduous legume tree. Tree, 19, 510–522.

Giesemann P, Eichenberg D, Stöckel M, Seifert LF, Gomes SIF, Merckx VSFT, Gebauer G. (2020) Dark septate endophytes and arbuscular mycorrhizal fungi (*Paris*-morphotype) affect the stable isotope composition of ‘classically’ non-mycorrhizal plants. Functional Ecology, 34, 2453–2466.

Haberer K, Grebenc T, Alexou M, Gessler A, Kraigher H, Rennenberg H. (2007) Effects of long-term free-air ozone fumigation on $\delta^{15}N$ and total N in *tagus sylvatica* and associated mycorrhizal fungi. Plant Biology, 9, 242–252.

Handa IT, Hagedorn F, Hättenschwiler S. (2008) No stimulation in root production in response to 4 years of in situ CO_2 enrichment at the Swiss treeline. Functional Ecology, 22, 348–358.

Hasselquist NJ, Santiago LS, Allen MF. (2010) Belowground nitrogen dynamics in relation to hurricane damage along a tropical dry forest chronosequence. Biogeochemistry, 98, 89–100.

Hayashi M, Caceres MLL, Nobori Y, Mijidsuren B, Boy J. (2018) Nitrogen isotope pattern in Mongolian larch stands at the southern Eurasian boreal forest boundary. Isotopes in Environmental and Health Studies, 54, 6.

Hobbie EA, Colpaert JV. (2003) Nitrogen availability and colonization by mycorrhizal fungi correlate with nitrogen isotope patterns in plants. New Phytologist, 157, 115–126.

Hobbie EA, Rygiewicz PT, Johnson MG, Moldenke AR. (2007) ^{13}C and ^{15}N in microarthropods reveal little response of Douglas-fir ecosystems to climate change. Global Change Biolgoy, 13, 1386–1397.

Hobbie EA, Colpaert JV, White MW, Ouimette AP, Macko SA. (2008) Nitrogen form, availability, and mycorrhizal colonization affect biomass and nitrogen isotope patterns in Pinus sylvestris. Plant and Soil, 310, 121–136.

Hobbie EA, Macko SA, Shugart HH. (1999) Insights into nitrogen and carbon dynamics of ectomycorrhizal and saprotrophic fungi from isotopic evidence. Oecologia, 118, 353–360.

Hobbie EA, Macko SA, Williams M. (2000) Correlations between foliar $\delta^{15}N$ and nitrogen concentrations may indicate plant-mycorrhizal interactions. Oecologia, 122, 273–283.

Hogberg P, Hogbon L, Schinkel H, Hogberg M, Johannisson C, Wallmark N. (1996) ^{15}N abundance of surface soils, roots and mycorrhizas in profiles of European forest soils. Oecologia, 108, 207–214.

Hooker TD, Stark JM, Norton U, Leffler AJ, Peek M, Ryel R. (2008) Distribution of ecosystem C and N within contrasting vegetation types in a semiarid rangeland in the Great Basin, USA. Biogeochemistry, 90, 291–308.

Hou EQ, Chen CR, Wen DZ, Kuang YW, Sun FF. (2015) Plant and soil $\delta^{13}C$ and $\delta^{15}N$ are linked to community biomass, litter production, and litter turnover rate in mature subtropical forests. Plant Ecology, 216, 859–872.

Hu CC, Liu XY. (2022) Plant nitrogen-use strategies and their responses to the urban elevation of atmospheric nitrogen deposition in southwestern China. Environmental Pollution, 311, 119969.

Hu CC, Liu XY, Yan YX, Lei YB, Tan YH, Liu CQ. (2022) A new isotope framework to decipher leaf-root nitrogen allocation and assimilation among plants in a tropical invaded ecosystem. Science of the Total Environment, 806, 151203.

Huber E, Wanek W, Gottfried M, Pauli H, Schweiger P, Arndt SK, Reiter K, Richter. (2007) Shift in soil-plant nitrogen dynamics of an alpine–nival ecotone. Plant Soil, 301, 65–76.

Hungate BA, Hart SC, Selmants PC, Boyle SI, Gehring CA. (2007) Soil responses to management, increased precipitation, and added nitrogen in ponderosa pine forests. Ecological Applications, 17, 1352–1365.

Huss-Danell K, Chaia E, Carlsson G. (2007) N_2 fixation and nitrogen allocation to above and below ground plant parts in red clover-grasslands. Plant and Soil, 299, 215–226.

Inglett PW, Reddy KR, Newman S, Lorenzen B. (2007) Increased soil stable nitrogen isotopic ratio following phosphorus enrichment: historical patterns and tests of two hypotheses in a phosphorus-limited wetland. Oecologial, 153, 99–109.

Isaac ME, Harmand JM, Drevon JJ. (2011) Growth and nitrogen acquisition strategies of *Acacia senegal* seedlings under exponential phosphorus additions. Journal of Plant Physiology, 168, 776–781.

Kitayama K, Iwamoto K. (2001) Patterns of natural ^{15}N abundance in the leaf-to-soil continuum of tropical rain forests differing in N availability on Mount Kinabalu, Borneo. Plant and Soil, 229, 203–212.

Klink S, Giesemann P, Hubmann T, Pausch J. (2020) Stable C and N isotope natural abundances of intraradical hyphae of arbuscular mycorrhizal fungi. Mycorrhiza, 30, 773–780.

Kolb KJ, Evans RD. (2002) Implications of leaf nitrogen recycling on the nitrogen isotope composition of deciduous plant tissues. New Phytologist, 156, 57–64.

Kudrin AA, Tsurikov SM, Tiunov AV. (2015) Trophic position of microbivorous and predatory soil nematodes in a boreal forest as indicated by stable isotope analysis. Soil Biology & Biochemistry, 86, 193–200.

Lindwall F, Vowles T, Ekblad A, Björk RG. (2013) Reindeer grazing has contrasting effect on species traits in *Vaccinium vitis-idaea* L. and *Bistorta vivipara* (L.) Gray. Acta Oecologica, 53, 33–37.

Liu CP, Yeh HW, She BH. (2006) N isotopes and N cycle in a 35-year-old plantation of the Guandaushi subtropical forest ecosystem, central Taiwan. Forest Ecology and Management, 235, 84–87.

Makarov MI, Buzin IS, Tiunov AV, Malyshev I, Kadulin MS, Korolev NE. (2019) Nitrogen isotopes in soils and plants of tundra ecosystems in the Khibiny Mountains. Eurasian Soil Science, 52(10) 1195–1206.

Marin-Spiotta E, Silver WL, Swanston CW, Ostertag R. (2009) Soil organic matter dynamics during 80 years of reforestation of tropical pastures. Global Change Biology, 15, 1584–1597.

Mayor JR, Schuur EAG, Mack MC, Hollingsworth TN, Ba˚a˚th E. (2012) Nitrogen isotope patterns in Alaskan Black Spruce reflect organic nitrogen sources and the activity of ectomycorrhizal fungi. Ecology, 15, 819–831.

Mayor JR, Wright SJ, Schuur EAG, Brooks ME, Turner BL. (2014) Stable nitrogen

isotope patterns of trees and soils altered by long-term nitrogen and phosphorus addition to a lowland tropical rainforest. Biogeochemistry, 119, 293–306.

Michelsen A, Quarmby C, Sleep D, Jonasson S. (1998) Vascular plant ^{15}N natural abundance in heath and forest tundra ecosystems is closely correlated with presence and type of mycorrhizal fungi in roots. Oecologia, 115, 406–418.

Moore TR, Alfonso A, Clarkson BR. (2018) Plant uptake of organic nitrogen in two peatlands. Plant and Soil, 433, 391–400.

Muhammad SA, Kadir MOAB, Rodhi AM, Hassan HM. (2017) Variations of $\delta^{15}C$ and $\delta^{15}N$ in oil palm tree organs: an insight into and N distribution. Journal of Oil Plant Research, 29, 242–250.

Murata K, Watermann F, Gonroudobou OBH, Hang LT, Yamanaka T, Lopez ML. (2022) Effect of black locust trees on the nitrogen dynamics of black pine trees in Shonai coastal forest, Japan. Plant and Soil, 474, 513–523.

Nadelhoffer KJ, Downs MR, Fry B. (1999) Sinks for ^{15}N-enriched additions to an oak forest and a red pine plantation. Ecological Applications, 9, 72–86.

Nadelhoffer KJ, Colman BP, Currie WS, MagillA, Aber JD. (2004) Decadal-scale fates of ^{15}N tracers added to oak and pine stands under ambient and elevated N inputs at the Harvard Forest (USA). Forest Ecology and Management, 196, 89–107.

Newingham BA, Vanier CH, Charlet TN, Ogle K, Smith SD, Nowak RS. (2013) No cumulative effect of 10 years of elevated [CO_2] on perennial plant biomass components in the Mojave Desert. Global Change Biology, 19(7) 2168–2181.

Nguyen TTN, Wallace HM, Xu CY, Xu ZH, Farrar MB, Joseph S, Zwieten LV, Bai SH. (2017) Short-term effects of organo-mineral biochar and organic fertilisers on nitrogen cycling, plant photosynthesis, and nitrogen use efficiency. Journal of Soil Sediments, 17, 2763–2774.

Nygren P, Leblanc HA. (2015) Dinitrogen fixation by legume shade trees and direct transfer of fixed N to associated cacao in a tropical agroforestry system. Tree Physiology, 35, 134–147.

Onipchenko VG, Makarov M, van Logtestijn RSP, Ivanov VB, Akhmetzhanova AA, Tekeev DK, Ermak AA, Salpagarova FS, Kozhevnikova AD, Cornelissen JHC. (2009) New nitrogen uptake strategy: specialized snow roots. Ecology Letters, 12, 758–764.

Ouimette A, Guo D, Hobbie E, Gu JC. (2013) Insights into root growth, function, and mycorrhizal abundance from chemical and isotopic data across root orders. Plant and Soil, 367, 313–326.

Pardo LH, Semaoune P, Schaberg PG, Eagar C, Sebilo M. (2012) Patterns in $\delta^{15}N$ in roots, stems, and leaves of sugar maple and American beech seedlings, saplings, and mature trees. Biogeochemistry, 112, 275–291.

Pellitier PT, Zak DR, Argiroff WA, Upchurch RA. (2021) Coupled shifts in ectomycorrhizal communities and plant uptake of organic nitrogen along a soil gradient: An isotopic perspective. Ecosystems, 24, 1976–1990.

Peuke AD, Gessler A, Rennenberg H. (2006) The effect of drought on C and N stable isotopes in different fractions of leaves, stems and roots of sensitive and tolerant

beech ecotypes. Plant Cell and Environment, 29, 823–835.

Piao HC, Li SL, Wang SJ, Li SH. (2017) The preference of nitrate uptake in Chinese prickly ash estimated by $\delta^{15}N$ values and cation concentrations. Environmental Earth Sciences, 76, 87.

Pocewicz A, Morgan P, Kavanagh K. (2007) The effects of adjacent land use on nitrogen dynamics at forest edges in northern Idaho. Ecosystems, 10, 226–238.

Ponette-González AG, Ewing HA, Fry M, Young KR. (2016) Soil and fine root chemistry at a tropical Andean timberline. Catena, 137, 350–359.

Pollierer MM, Langel R, Scheu S, Maraun M. (2009) Compartmentalization of the soil animal food web as indicated by dual analysis of stable isotope ratios ($^{15}N/^{14}N$ and $^{13}C/^{12}C$). Soil Biology & Biochemistry, 41, 1221–1226.

Posada JM, Schuur EAG. (2011) Relationships among precipitation regime, nutrient availability, and carbon turnover in tropical rain forests. Oecologia, 165, 783–795.

Rivero-Villar A, Templer PH, Parra-Tabla Victor, Campo J. (2018) Differences in nitrogen cycling between tropical dry forests with contrasting precipitation revealed by stable isotopes of nitrogen in plants and soils. Biotropica, 50, 859–867.

Savard MM, Martineau C, Laganière J, Bégin C, Mariona J, Smirnoff A, Stefani F, Bergeron J, Rheault K, Paré D, Séguin A. (2021) Nitrogen isotopes in the soil-to-tree continuum — Tree rings express the soil biogeochemistry of boreal forests exposed to moderate airborne emissions. Science of the Total Environment, 780, 146581.

Schmidt S, Stewart GR. (2003) $\delta^{15}N$ values of tropical savanna and monsoon forest species reflect root specialisations and soil nitrogen status. Oecologia, 134, 569–577.

Schleppi P, Bucher-Wallin I, Hagedorn F, Korner C. (2012) Increased nitrate availability in the soil of a mixed mature temperate forest subjected to elevated CO_2 concentration (canopy FACE). Global Change Biology, 18, 757–768.

Seidel F, Lopez C ML, Oikawa A, Yamanaka T. (2019) Seasonal nitrogen partitioning in Japanese cedar (*Cryptomeria japonica*, D. Don) tissues. Plant and Soil, 442, 511–529.

Seidel F, Lopez C ML, Celi L, Bonifacio E, Oikawa A, Yamanaka T. (2019) N isotope fractionation in tree Tissues during N reabsorption and remobilization in *fagus crenata* Blume. Forests, 10(4) 330.

Sheng ZL, Huang YM, He KJ, Borjigin N, Yang HY, Chen HY, Li EG, Xu X, Duan L. (2018) Responses of plant ^{15}N natural abundance and isotopic fractionation to N addition reflect the N status of a temperate steppe in China. Journal of Plant Ecology, 12, 550–563.

Soper FM, Richards AE, Siddique I, Aidar MPM, Cook GD, Hutley LB, Robinson N, Schmidt S. (2015) Natural abundance ($\delta^{15}N$) indicates shifts in nitrogen relations of woody taxa along a savanna–woodland continental rainfall gradient. Oecologia, 178, 297–308.

Song WC, Tong XJ, Liu YH, Li WK. (2020) Microbial community, newly sequestered soil organic carbon, and $\delta^{15}N$ variations driven by tree roots. Frontiers in Microbiology, 11, 314.

Stock WD, Evans JR. (2006) Effects of water availability, nitrogen supply and atmospheric CO_2 concentrations on plant nitrogen natural abundance values. Functional Plant Biology, 33, 219–227.

Tatsumi C, Hyodo F, Taniguchi T, Shi WY, Koba K, Fukushima K, Du S, Yamanaka N, Templer P, Tateno R. (2021) Arbuscular mycorrhizal community in roots and nitrogen uptake patterns of understory trees beneath ectomycorrhizal and non-ectomycorrhizal overstory Trees. Frontiers in Plant Science, 11, 583585.

Tedersoo L, Naadel T, Bahram M, Pritsch K, Buegger F, Leal M, Koljalg U, Poldmaa K. (2012) Enzymatic activities and stable isotope patterns of ectomycorrhizal fungi in relation to phylogeny and exploration types in an afrotropical rain forest. New Phytologist, 195, 832–843.

Templer PH, Weathers KC, Ewing HA, Dawson TE, Mambelli S, Lindsey AM, Webb J, Boukili VK. Firestone MK. (2015) Fog as a source of nitrogen for redwood trees: evidence from fluxes and stable isotopes. Journal of Ecology, 103, 1397–1407.

Templer PH, Arthur MA, Lovett GM, Weathers KC. (2007) Plant and soil natural abundance $\delta^{15}N$: indicators of relative rates of nitrogen cycling in temperate forest ecosystems. Oecologia, 153, 399–406.

Vaario LM, Sah SP, Norisada M, Narimatsu M, Matsushita N. (2018) Tricholoma matsutake may take more nitrogen in the organic form than other ectomycorrhizal fungi for its sporocarp development: the isotopic evidence. Mycorhiza, 29, 51–59.

Voronina PY, Mukhinb VA, Velivetskayac TA, Ignatevc AV, Kuznetsova VlV. (2017) Isotope composition of carbon and nitrogen in tissues and organs of *betula pendula*. Russtan Journal of Plant Phystology, 64, 127–132.

Wang LX, Macko SA. (2011) Constrained preferences in nitrogen uptake across plant species and environments. Plant Cell & Environment, 34, 525–534.

Wang H, Hu GQ, Luo YH, Su ZH, Zhuge YP, Meng FQ. (2016) Responses of natural ^{15}N abundance in cauliflower (*Brassica oleracea* L. var. botrytis) and soil to the application of organic and chemical fertilizers. Canadian journal of plant science, 96, 819–827.

Wang Y, Schimel JP, Nisbet RM, Gardea-Torresdey JL, Holden PA. (2020) Soybeans grown with carbonaceous nanomaterials maintain nitrogen stoichiometry by assimilating soil nitrogen to offset impaired dinitrogen fixation. ACS Nano, 14, 585–594.

Williams MA, Rice CW, Owensby CE. (2006) Natural ^{15}N abundances in a tallgrass prairie ecosystem exposed to 8-y of elevated atmospheric CO_2. Soil Biology & Biochemistry, 38. 409–412.

Yan GY, Han SJ, Zhou MX, Sun WJ, Huang BB, Wang HL, Xing YJ, Wang GG. (2020) Variations in the natural ^{13}C and ^{15}N abundance of plants and soils under long-term N addition and precipitation reduction: interpretation of C and N dynamics. Forest Ecosystems, 7, 49.

Yang LQ, He TG, Yu YF, Li ZY, Li DJ. (2017) Community-wide consistence in plant N acquisition during post-agricultural succession in a karst area, southwest China. New Forests, 49, 197–214.

Ye L, Abbadie L, Bardoux G, Lata JC, Nacro HB, Masse D, de Parseval H, Barot S. (2015) Contrasting impacts of grass species on nitrogen cycling in a grazed Sudanian savanna. Acta Oecologica, 63, 8–15.

Zeng XH, Ni ZY, Diao HX, Jiang K, Hu C, Shao L, Huang WC. (2021) Root endophytic fungal community and carbon and nitrogen stable isotope patterns differ among *Bletilla* species (Orchidaceae). Journal of Fungi, 7, 69.

Zeller B, Brechet C, Maurice JP, Tacon FL. (2008) Saprotrophic versus symbiotic strategy during truffle ascocarp development under holm oak. A response based on ^{13}C and ^{15}N natural abundance. Annals of Forest Science. 65, 607.

Zhang JX, Gu LH, Zhang JB, Wu RN, Wang F, Lin GH, Wu B, Lu Q, Meng P. (2017) The interaction between nitrogen and phosphorous is a strong predictor of intra-plant variation in nitrogen isotope composition in a desert species. Biogeosciences, 14, 131–144.

Zhao W, Guo B, Liang KH, Wang DH, Yang SM. (2017) Physiological, elemental, and stable isotope responses of the organs of mungbean to reduced atmospheric pressure. In Vitro Cellular and Developmental Biology, 53, 113–121.

Zhou Y, Mushinski RM, Hyodo A, Wu B, Boutton TW. (2018) Vegetation change alters soil profile $\delta^{15}N$ values at the landscape scale. Soil Biology and Biochemistry, 119, 110–120.

Zhu FF, Dai LM, Hobbie EA, Koba K, Liu XY, Gurmesa GA, Huang SN, Li SL, Li YH, Han SJ, Fang YT. (2019) Uptake patterns of glycine, ammonium, and nitrate differ among four common tree species of northeast China. Frontiers in Plant Science, Frontiers in Plant Science, 10, 799.

Supplementary Text 3 | Publications of $\delta^{15}N$ observations for soil EON, NH_4^+ and NO_3^-.

Boddey RM, Peoples MB, Palmer B, Dart PJ. (2000) Use of the ^{15}N natural abundance technique to quantify biological nitrogen fixation by woody perennials. Nutrient Cycling in Agroecosystems, 57, 235–270.

Bourgeois I, Clement JC, Caillon N, Savarino J. (2019) Foliar uptake of atmospheric nitrate by two dominant subalpine plants: insights from in situ triple-isotope analysis. New Phytologist, 223, 1784–1794.

Brearley FQ. (2013) Nitrogen stable isotopes indicate differences in nitrogen cycling between two contrasting Jamaican montane forests. Plant and Soil, 367, 465–476.

Buzek F, Cejkova B, Jackova I, Kram P, Oulehle F, Myska O, Curik J, Veselovsky F, Novak M. (2020) ^{15}N study of the reactivity of atmospheric nitrogen in four mountain forest catchments (Czech Republic, central Europe). Applied Geochemistry, 116, 104567.

Cheng SL, Fang HJ, Yu GR, Zhu TH, Zheng JJ. (2010) Foliar and soil ^{15}N natural abundances provide field evidence on nitrogen dynamics in temperate and boreal forest ecosystems. Plant and Soil, 337, 285–297.

Dynarski KA, Houlton BZ. (2020) Isotopic constraints on plant nitrogen acquisition

strategies during ecosystem retrogression. Oecologia, 192, 603–614.

Fang YT, Koba K, Makabe A, Takahashi C, Zhu WX, Hayashi T, Hokari AA, Urakawad R, Bai E, Houlton BZ, Xi D, Zhang SS, Matsushita K, Tu Y, Liu DW, Zhu FF, Wang ZY, Zhou GY, Cheng DX, Makita T, Toda H, Liu XY, Chen QS, Zhang DQ, Li YD, Yoh M. (2015) Microbial denitrification dominates nitrate losses from forest ecosystems. Proceedings of the National Academy of Sciences of the United States of America, 112(5) 1470–1474.

Gurmesa GA, Hobbie EA, Zhang SS, Wang A, Zhu FF, Zhu WX, Koba K, Yoh M, Wang CK, Zhang QL, Fang YT. (2022) Natural ^{15}N abundance of ammonium and nitrate in soil profiles: New insights into forest ecosystem nitrogen saturation. Ecosphere, 13, e3998.

Hemsley TL, MacKenzie MD, Quideau SA. (2019) Ecophysiological response of aspen (*Populus tremuloides*) and jack pine (*Pinus banksiana*) to atmospheric nitrogen deposition on reconstructed boreal forest soils in the Athabasca oil sands region. Science of the Total Environment, 696, 133544.

Hobbie EA, Macko SA, Williams M. (2000) Correlations between foliar δ^{15}N and nitrogen concentrations may indicate plant-mycorrhizal interactions. Oecologia, 122, 273–283.

Houlton BZ, Sigman DM, Schuur EAG, Hedin LO. (2007) A climate-driven switch in plant nitrogen acquisition within tropical forest communities. Proceedings of the National Academy of Sciences of the United States of America, 104, 8902–8906.

Hu CC, Lei YB, Tan YH, Sun XC, Xu H, Liu CQ, Liu XY. (2019) Plant nitrogen and phosphorus utilization under invasive pressure in a montane ecosystem of tropical China. Journal of Ecology, 107, 372–386.

Hu CC, Liu XY. (2022) Plant nitrogen-use strategies and their responses to the urban elevation of atmospheric nitrogen deposition in southwestern China. Environmental Pollution, 311, 119969.

Hobbie EA, Macko SA, Shugart HH. (1999) Insights into nitrogen and carbon dynamics of ectomycorrhizal and saprotrophic fungi from isotopic evidence. Oecologia, 118, 353–360.

Hungate BA, Hart SC, Selmants PC, Boyle SI, Gehring CA. (2007) Soil responses to management, increased precipitation, and added nitrogen in ponderosa pine forests. Ecological Applications, 17(5) 1352–1365.

Huygens D, Díaz S, Urcelay C, Boeckx P. (2016) Microbial recycling of dissolved organic matter confines plant nitrogen uptake to inorganic forms in a semi-arid ecosystem. Soil Biology & Biochemistry, 101, 142–151.

Inglett PW, Reddy KR, Newman S, Lorenzen B. (2007) Increased soil stable nitrogen isotopic ratio following phosphorus enrichment: historical patterns and tests of two hypotheses in a phosphorus-limited wetland. Oecologia, 153, 99–109.

Kang XQ, Niu Y, Yu Y, Guo P, Hou QY, Lu XF, Wu YL. (2022) Effect of rainfall-runoff process on sources and transformations of nitrate using a combined approach of dual isotopes, hydrochemical and Bayesian model in the Dagang River basin. Science of the Total Environment, 837, 155674.

Koba K, Isobe K, Takebayashi Y, Fang YT, Sasaki Y, Saito W, Yoh M, Mo J, Liu L, Lu X, Zhang T, Zhang W, Senoo K. (2010) $\delta^{15}N$ of soil N and plants in a N-saturated subtropical forest of southern China. Rapid Communication in Mass Spectrometry, 24, 2499–2506.

Koba K, Tokuchi N, Yoshioka T, Hobble EA, Iwatsubo G. (1998) Natural Abundance of Nitrogen-15 in a Forest Soil. Soil Science Society of America Journal, 62, 778–781.

Kwak JH, Choi WJ, Lim SS, Lee SH, Lee SM, Chang SX, Jung JW, Yoon KS, Choi SM. (2008) Sources and transformations of N in reclaimed coastal tidelands: evidence from soil $\delta^{15}N$ data. Environmental Geology, 53, 1331–1338.

Liu XY, Koba K, Makabe A, Li XD, Yoh M, Liu CQ. (2013) Ammonium first: natural mosses prefer atmospheric ammonium but vary utilization of dissolved organic nitrogen depending on habitat and nitrogen deposition. New Phytologist, 199, 407–419.

Makarov MI, Buzin IS, Tiunov AV, Malyshev I, Kadulin MS, Korolev NE. (2019) Nitrogen isotopes in soils and plants of tundra ecosystems in the Khibiny Mountains. Eurasian Soil Science, 52(10) 1195–1206.

Makarov MI, Malysheva TI, Menyailo O V. (2019) Isotopic composition of nitrogen and transformation of nitrogen compounds in meadow-alpine soils. Eurasian Soil Science, 52(9) 1028–1037.

Makarov MI, Malysheva TI, Cornelissen JHC, van Logtestijn RSP, GlasserB. (2008) Consistent patterns of ^{15}N distribution through soil profiles in diverse alpine and tundra ecosystems. Soil Biology & Biochemistry, 40, 1082–1089.

Mayor JR, Schuur EAG, Mack MC, Hollingsworth TN, Baath E. (2012) Nitrogen isotope patterns in Alaskan Black Spruce reflect organic nitrogen sources and the activity of ectomycorrhizal fungi. Ecology, 15, 819–831.

Mayor JR, Wright SJ, Schuur EAG, Brooks ME, Turner BL. (2014) Stable nitrogen isotope patterns of trees and soils altered by long-term nitrogen and phosphorus addition to a lowland tropical rainforest. Biogeochemistry, 119, 293–306.

Nygren P, Leblanc HA. (2015) Dinitrogen fixation by legume shade trees and direct transfer of fixed N to associated cacao in a tropical agroforestry system. Tree Physiology, 35, 134–147.

Pellitier PT, Zak DR, Argiroff WA, Upchurch RA. (2021) Coupled shifts in ectomycorrhizal communities and plant uptake of organic nitrogen along a soil gradient: An isotopic perspective. Ecosystems, 24, 1976–1990.

Portl K, Zechmeister-Boltenstern S, Wanek W, Ambus P, Berger TW. (2007) Natural ^{15}N abundance of soil N pools and N_2O reflect the nitrogen dynamics of forest soils. Plant and Soil, 295, 79–94.

Qin Y, Zhang D, Wang FS. (2019) Using nitrogen and oxygen isotopes to access sources and transformations of nitrogen in the Qinhe Basin, North China. Environmental Science and Pollution Research, 26, 738–748.

Ren HY, Tian L, Zhu Y, Xu ZW, Zeng DH, Fang YT, Han GD. (2022) Nitrogen and water addition alter nitrogen uptake preferences of two dominant plant species in a

typical Inner Mongolian steppe. Chinese Science Bulletin, 67, 1459–1468.

Rummel PS, Well R, Pausch J, Pfeiffer B, Dittert K. (2021) Carbon availability and nitrogen mineralization control denitrification rates and product stoichiometry during initial maize litter decomposition. Applied Science, 11, 5309.

Savard MM, Martineau C, Laganière J, Bégin C, Mariona J, Smirnoff A, Stefani F, Bergeron J, Rheault K, Paré D, Séguin A. (2021) Nitrogen isotopes in the soil-to-tree continuum — Tree rings express the soil biogeochemistry of boreal forests exposed to moderate airborne emissions. Science of the Total Environment, 780, 146581.

Schimann H, Ponton S, Hattenschwiler S, Ferryc B, Lensi R, Domenach AM, Roggy JC. (2008) Differing nitrogen use strategies of two tropical rainforest late successional tree species in French Guiana: Evidence from ^{15}N natural abundance and microbial activities. Soil Biology & Biochemistry, 40, 487–494.

Takebayashi Y, Koba K, Sasaki Y, Fang YT, Yoh M. (2010) The natural abundance of ^{15}N in plant and soil-available N indicates a shift of main plant N resources to NO_3^- from NH_4^+ along the N leaching gradient. Rapid Communication in Mass Spectrometry, 24, 1001–1008.

Tang B, Man J, Bai YF. Leaf nitrogen acquisition of *Leymus chinensis* varies with leaf age and land use change in a semiarid grassland. Environmental and Experimental Botany, 175, 104051.

Tateno R, Nakayama M, Yano M, Fukuzawa K, Inagaki Y, Koba K, Ugawa S. (2020) Nitrogen source utilization in co-existing canopy tree and dwarf bamboo in a northern hardwood forest in Japan. Trees, 34, 1047–1057.

Tatsumi C, Hyodo F, Taniguchi T, Shi WY, Koba K, Fukushima K, Du S, Yamanaka N, Templer P, Tateno R. (2021) Arbuscular mycorrhizal community in roots and nitrogen uptake patterns of understory trees beneath ectomycorrhizal and non-ectomycorrhizal overstory trees. Frontiers in Plant Science, 11, 583585.

Wang J, Wen XF, Lyu SD, Zhang XY, Li SG, Guo QJ. (2021) Vegetation recovery alters soil N status in subtropical karst plateau area: Evidence from natural abundance $\delta^{15}N$ and $\delta^{18}O$. Plant and Soil, 460, 609–623.

Wang RZ, Penuelas J, Li T, Liu HY, Wu H, Zhang YG, Sardans J, Jiang Y. Natural abundance of ^{13}C and ^{15}N provides evidence for plant–soil carbon and nitrogen dynamics in a N-fertilized meadow. Ecology, 102(2) e03348.

Weintraub SR, Brooks PD, Bowen GJ. (2017) Interactive effects of vegetation type and topographic position on nitrogen availability and loss in a temperate montane ecosystem. Ecosystems, 20, 1073–1088.

Yang LQ, He TG, Yu YF, Li ZY, Li DJ. (2017) Community-wide consistence in plant N acquisition during post-agricultural succession in a karst area, southwest China. New Forests, 49, 197–214.

Zhang ZL, Li N, Xiao J, Zhao CZ, Zou TT, Li DD, Liu Q, Yin HJ. (2018) Changes in plant nitrogen acquisition strategies during the restoration of spruce plantations on the eastern Tibetan Plateau, China. Soil Biology and Biochemistry, 119, 50–58.

Zhou XL, Wang A, Hobbie E, Zhu FF, Wang XY, Li YH, Fang YT. (2021) Nitrogen uptake strategies of mature conifers in Northeastern China, illustrated by the ^{15}N

natural abundance method. Ecological Processes, 10, 36.

Zhu FF, Dai LM, Hobbie EA, Koba K, Liu XY, Gurmesa GA, Huang SN, Li SL, Li YH, Han SJ, Fang YT. (2019) Uptake patterns of glycine, ammonium, and nitrate differ among four common tree species of northeast China. Frontiers in Plant Science, 10, 799.

Supplementary Text 4 | Publications of fractional contributions of different N species to non-leguminous terrestrial plants based on different methods.

1. Chapin III FS, Moilanen L, Kielland K. (1993) Preferential use of organic nitrogen for growth by a non-mycorrhizal arctic sedge. Nature, 361, 150–153.
2. Näsholm T. Ekblad A, Nordin A, Giesler R, Hogberg M, Hogberg P. (1998) Boreal forest plants take up organic nitrogen. Nature, 392, 914–916.
3. Öhlund J, Näsholm T. (2001) Growth of conifer seedlings on organic and inorganic nitrogen sources. Tree Physiology, 21, 1319–1326.
4. Liu XY, Koba K, Koyama LA, et al. (2018) Nitrate is an important nitrogen source for Arctic tundra plants. Proceedings of the National Academy of Sciences of the United States of America, 115, 3398–3403.
5. Nordin A, Högberg P, Näsholm T. (2001) Soil nitrogen form and plant nitrogen uptake along a boreal forest productivity gradient. Oecologia, 129, 125–132.
6. Guo WJ, Zhang ZL, Liu Q, Xiao J, Yin HJ. (2021) Seasonal variations in plant nitrogen acquisition in an ectomycorrhizal alpine forest on the eastern Tibetan Plateau, China. Plant Soil, 459, 79–91.
7. Mckane RB, Johnson LC, Shaver GR, Nadelhoffer KJ, Rastetter EB, Fry B, Giblin AE, Kielland K, Kwiatkowski BL, Laundre JA, Murray G. (2002) Resource-based niches provide a basis for plant species diversity and dominance in arctic tundra. Nature, 415, 68–71.
8. Xu XL, Ouyang H, Cao GM, Richter A, Wanek W, Kuzyakov Y. (2011) Dominant plant species shift their nitrogen uptake patterns in response to nutrient enrichment caused by a fungal fairy in an alpine meadow. Plant Soil, 341, 495–504.
9. Zhu FF, Dai LM, Hobbie EA, Koba K, Liu XY, Gurmesa GA, Huang SN, Li SL, Li YH, Han SJ, Fang YT. (2019) Uptake patterns of glycine, ammonium, and nitrate differ among four common tree species of Northeast China. Frontiers in Plant Science, 10, 799.
10. Cao JR, Yang LY, Pang S, Yang JJ, Hu Y, Li YC, Li LH, Wang QB. (2021) Convergent nitrogen uptake patterns and divergent nitrogen acquisition strategies of coexisting plant species in response to long-term nitrogen enrichment in a temperate grassland. Environmental and Experimental Botany, 185, 104412.
11. Zhou XL, Wang A, Hobbie E, Zhu FF, Wang XY, Li YH, Fang YT. (2021) Nitrogen uptake strategies of mature conifers in Northeastern China, illustrated by the ^{15}N natural abundance method. Ecological Processes, 10, 36.
12. Huang YL, Du LS, Lei YB, Liang JY. (2023) Nitrogen preference of dominant

species during Hailuogou Glacier retreat succession on the Eastern Tibetan Plateau. Plants, 12, 838.

13. Engelbrecht Clemmensen K, Lærkedal Sorensen P, Michelsen A, Jonasson S, Strom L. (2008) Site-dependent N uptake from N-form mixtures by arctic plants, soil microbes and ectomycorrhizal fungi. Oecologia, 155, 771–783.

14. Volder A, Bliss LC, Lambers H. (2000) The influence of temperature and nitrogen source on growth and nitrogen uptake of two polar-desert species, *Saxifraga caespitosa* and *Cerastium alpinum*. Plant and soil, 227, 139–148.

15. Gao JQ, Mo Y, Xu XL, Zhang XW, Yu FH. (2014) Spatiotemporal variations affect uptake of inorganic and organic nitrogen by dominant plant species in an alpine wetland. Plant Soil, 381, 271–278.

16. Li CC, Li QR, Qiao N, Xu XL, Li QK, Wang HM. (2015) Inorganic and organic nitrogen uptake by nine dominant subtropical tree species. iForest, 9, 253–258.

17. Wittich B, Homeier J, Leuschner C. (2015) Ammonium, nitrate and glycine uptake of six Ecuadorian tropical montane forest tree species: an *in situ* pot experiment with saplings. Journal of Tropical Ecology, 31, 139–152.

18. Huygens D, Diaz S, Urcelay C, Boeckx P. (2016) Microbial recycling of dissolved organic matter confines plant nitrogen uptake to inorganic forms in a semi-arid ecosystem. Soil Biology & Biochemistry, 101, 142–151.

19. Houlton BZ, Sigman DM, Schuur EAG, Hedin LO. (2007) A climate-driven switch in plant nitrogen acquisition within tropical forest communities. Proceedings of the National Academy of Sciences of the United States of America, 104, 8902–8906.

20. Andersen KM, Mayor JR, Turner BL. (2017) Plasticity in nitrogen uptake among plant species with contrasting nutrient acquisition strategies in a tropical forest. Ecology, 98, 1388–1398.

21. Takebayashi Y, Koba K, Sasaki Y, Fang YT, Yoh M. (2010) The natural abundance of ^{15}N in plant and soil-available N indicates a shift of main plant N resources to NO_3^- from NH_4^+ along the N leaching gradient. Rapid Communications in Mass Spectrometry, 24, 1001–1008.

22. Andersen KM, Turner BL. (2013) Preferences or plasticity in nitrogen acquisition by understorey palms in a tropical montane forest. Journal of Ecology, 101, 819–825.

23. Jin VL, Evans RD. (2010) Elevated CO_2 increases plant uptake of organic and inorganic N in the desert shrub Larrea tridentata. Oecologia, 163, 257–266.

24. Kahmen A, Livesley SJ, Arndt SK. (2009) High potential, but low actual, glycine uptake of dominant plant species in three Australian land-use types with intermediate N availability. Plant Soil, 325, 109–121.

25. Liu M, Li CC, Xu XL, Wanek W, Jiang N, Wang HM, Yang XD. (2017) Organic and inorganic nitrogen uptake by 21 dominant tree species in temperate and tropical forests. Tree Physiology, 37, 1515–1526.

26. Ruess RW. (1988) The interaction of defoliation and nutrient uptake in

Sporobolus kentrophyllus, a short-grass species from the Serengeti Plains. Oecologial, 77, 550–556.

27. Averill C, Finzi A. (2011) Increasing plant use of organic nitrogen with elevation is reflected in nitrogen uptake rates and ecosystem $\delta^{15}N$. Ecology, 92, 883–891.
28. Scott EE, Rothstein DE. (2011) Amino acid uptake by temperate tree species characteristic of low- and high-fertility habitats. Oecologia, 167, 547–557.
29. Zhang ZL, Li N, Xiao J, Zhao CZ, Zou TT, Li DD, Liu Q, Yin HJ. (2018) Changes in plant nitrogen acquisition strategies during the restoration of spruce plantations on the eastern Tibetan Plateau, China. Soil Biology and Biochemistry, 119, 50–58.
30. Wilkinson A, Hill PW, Vaieretti MV, Farrar JF, Jones DL, Bardgett RD. (2015) Challenging the paradigm of nitrogen cycling: no evidence of in situ resource partitioning by coexisting plant species in grasslands of contrasting fertility. Ecology and Evolution, 5(2): 275–287.
31. Mead DJ, Preston CM. (1994) Distribution and retranslocation of ^{15}N in lodgepole pine over eight growing seasons. Tree Physiology, 14, 389–402.
32. Bennett JN, Prescott CE. (2004) Organic and inorganic nitrogen nutrition of western red cedar, western hemlock and salal in mineral N-limited cedar–hemlock forests. Oecologia, 141, 468–476.
33. Zhuang W, Wang M, Xiao Y, Wu N. (2022) Differential uptake of nitrogen forms by two herbs in the Gurbantunggut desert, Central Asia. Plant Biology, 24, 758–765.
34. Kastovská E, Santrucková H. (2011) Comparison of uptake of different N forms by soil microorganisms and two wet-grassland plants: A pot study. Soil Biology & Biochemistry, 43, 1285–1291.
35. Felten SV, Hector A, Buchmann N, Niklaus PA, Schmid B, Scherer-Lorenzen M. (2009) Belowground nitrogen partitioning in experimental grassland plant communities of varying species richness. Ecology, 90, 1389–1399.
36. Cheng XM, Bledsoe CS. (2004) Competition for inorganic and organic N by blue oak (*Quercus douglasii*) seedlings, an annual grass, and soil microorganisms in a pot study. Soil Biology & Biochemistry, 36, 135–144.

Supplementary Text 5｜Publications of effects of increasing N deposition, temperature, and precipitation on fractional contributions of different N species to non-leguminous terrestrial plants.

1. Zhou MX, Yan GY, Xing YJ, Chen F, Zhang X, Wang JY, Zhang JH, Dai GH, Zheng XB, Sun WJ, Wang QG, Liu T. (2019) Nitrogen deposition and decreased precipitation does not change total nitrogen uptake in a temperate forest. Science of the Total Environment, 651, 32–41.
2. Cao JR, Yang LY, Pang S, Yang JJ, Hu Y, Li YC, Li LH, Wang QB. (2021) Convergent nitrogen uptake patterns and divergent nitrogen acquisition strategies of coexisting plant species in response to long-term nitrogen enrichment in a

temperate grassland. Environmental and Experimental Botany, 185, 104412.

3. Mayor JR, Wright JS, Schuur EAG, Brooks ME, Turner BL. (2014) Stable nitrogen isotope patterns of trees and soils altered by long-term nitrogen and phosphorus addition to a lowland tropical rainforest. Biogeochemistry, 119, 293–306.

4. Boczulak SA, Hawkins BJ, Roy R. (2014) Temperature effects on nitrogen form uptake by seedling roots of three contrasting conifers. Tree Physiology, 34, 513–523.

5. Houlton BZ, Sigman DM, Schuur EAG, Hedin LO. (2007) A climate-driven switch in plant nitrogen acquisition within tropical forest communities. Proceedings of the National Academy of Sciences of the United States of America, 104, 8902–8906.
